# Diversity of secoiridoid glycosides in leaves of UK and Danish ash provide new insight for ash dieback management

**DOI:** 10.1038/s41598-020-76140-z

**Published:** 2020-11-11

**Authors:** John D. Sidda, Lijiang Song, Jack L. Parker, David J. Studholme, Christine Sambles, Murray Grant

**Affiliations:** 1 School of Life Sciences, University of Warwick, Coventry, CV4 7AL UK; 2 Department of Chemistry, University of Warwick, Coventry, CV4 7AL UK; 3 School of Biosciences, University of Exeter, Exeter, EX4 4QD UK

**Keywords:** Chemical ecology, Cheminformatics, Natural products, Small molecules

## Abstract

Secoiridoid glycosides are anti-feeding deterrents of the *Oleaceae* family recently highlighted as potential biomarkers in Danish ash trees to differentiate between those tolerant and susceptible to the fungal disease ash dieback. With the knowledge that emerald ash borer has recently entered Europe from Russia, and that extensive selection trials are ongoing in Europe for ash dieback tolerant European ash (*Fraxinus excelsior*), we undertook comprehensive screening of secoiridoid glycosides in leaf extracts of trees tolerant and susceptible to ash dieback sampled from sites in the UK and Denmark. Here we report an unexpected diversity of secoiridoid glycosides in UK trees and higher levels of secoiridoid glycosides in the UK sample group. While it is unlikely that secoiridoid glycosides generally can serve as reliable markers for ash dieback susceptibility, there are differences between tolerant and susceptible groups for specific secoiridoids. We predict that the high levels—and structural diversity—of secoiridoids present in the UK group may provide a robust reservoir of anti-feeding deterrents to mitigate future herbivore threats such as the Emerald ash borer.

## Introduction

**Figure 1:**
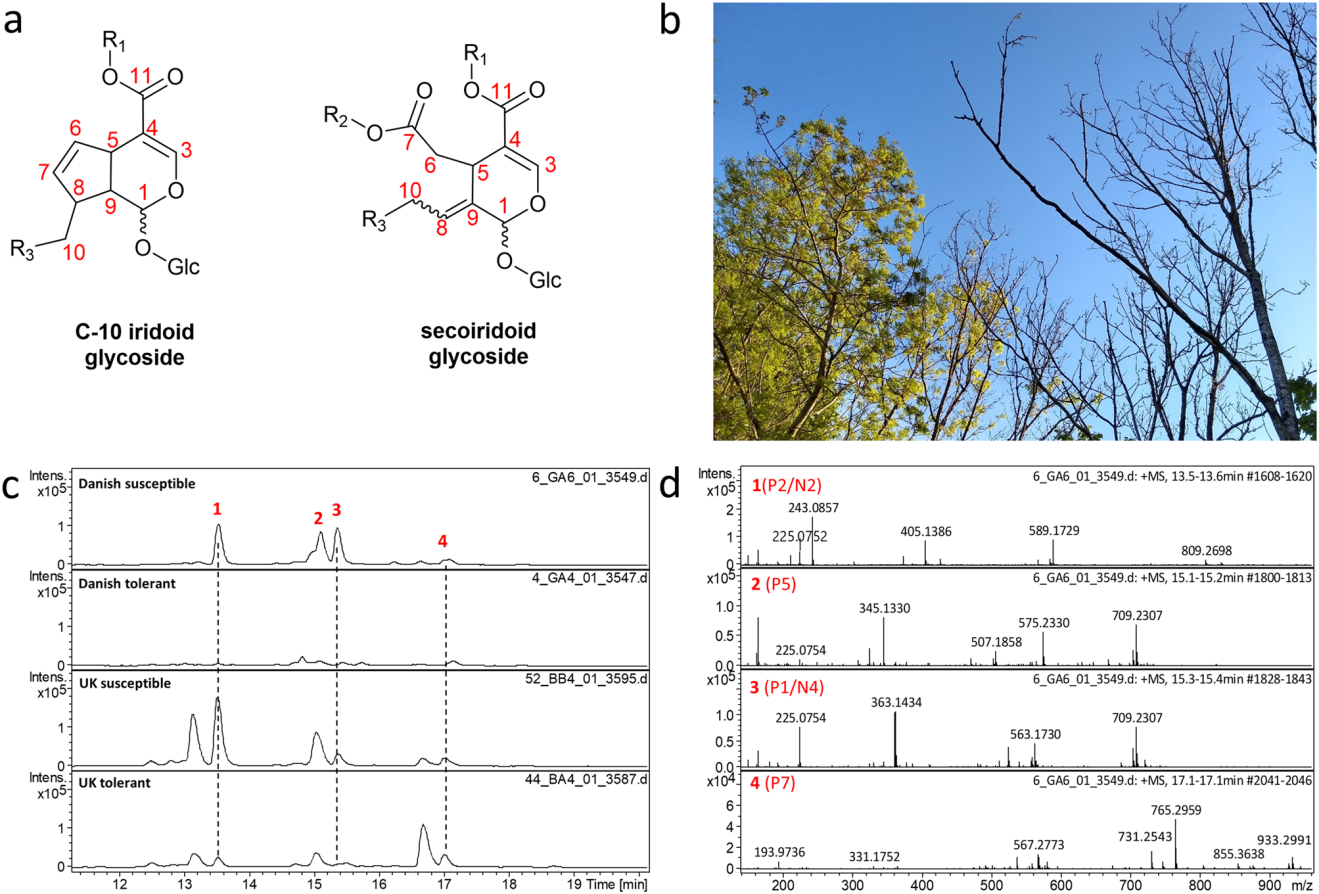
Summary of secoiridoid derived compounds previously identified from metabolite profiling. (**a**) General core structure of iridoid glycosides and secoiridoid glycosides (**b**) *Fraxinus excelsior* affected by ash dieback (mid-Devon, UK). (**c**) Extracted ion chromatograms *m/z* 589.1732 (**1**/P2/N2); 709.2315 (**2**/P5 and **3**/P1/N4); 933.3007 (**4**/P7) representing [M+Na]^+^ adducts of compounds previously identified as markers of susceptibility to ADB in Danish ash^27^. (**d**) Positive ion mode mass spectra of **1**–**4**.

Iridoid glycosides are a large class of natural products prevalent in the plant kingdom. Derived from the monterpenoid iridotrial, they can be broadly divided into two subclasses: iridoids, containing an intact cyclopentene ring, and secoiridoids^1–3^. Predominantly, the iridoid glycosides identified in the *Oleaceae* family, which ash (*Fraxinus*) and olive (*Olea*) genera belong to, are secoiridoids. Secoiridoids are characterised by a 10-carbon core skeleton in which the bond between C-7 and C-8 in the cyclopentene ring is cleaved, generating carboxylic acid and olefin moieties (Fig. 1)^1,2,4^. Substitutions of the secoiridoid core typically occur on the two carboxylic acid groups located at C7 and C11, in addition to glycosylation (usually glucose) on C1 of the iridoid core. Further oxidations may occur at C-10^2^. The secoiridoid glycoside biosynthetic pathways have been partially elucidated in the Madagascar periwinkle (*Catharanthus roseus*) and olive (*Olea europaea*)^5,6^. Many secoiridoid glycosides have previously been isolated from ash leaves (*Fraxinus* genus) including oleuropein, nuzhenide, excelsioside, GL3 and GL5^7,8^.

Numerous biological activities for secoiridoid glycosides have been reported, including antioxidant activity associated with tyrosol and hydroxytyrosol groups^9–12^. Oleuropein, a major constituent of olive oil, has modest antibiotic activity and has been reported to stabilise α-synuclein, aggregation of which is a key step in the development of Parkinson’s disease^13,14^. There are numerous reports of aglycones and dialdehyde derivatives of oleuropein and ligustroside (ligstroside) acting as anti-feeding molecules^15,16^. Interestingly, secoiridoid glycoside biosynthetic genes are wound induced in common Centaury (*Centaurium erythraea* Rafn)^17^, and systemic responses to wounding and herbivory strongly overlap^18^.

European ash is currently under threat from ash dieback (ADB) caused by the fungus *Hymenoscyphus fraxineus* which has devastated ash across mainland Europe over the past 25 years^19,20^. ADB was first reported in the UK in 2012, though recent reports suggest *H. fraxineus* arrived as early as the late 1990s^21^. Meanwhile, across North America, the Emerald ash borer (EAB; *Agrilus planipennis*) has destroyed millions of trees of native ash species. EAB is currently found in Russia and Ukraine is likely to become a major pest in Europe^22–24^.

Whilst there has been extensive effort to identify robust DNA molecular markers for ADB tolerance or susceptibility, observation of disease states in the field is critical to provide the necessary germplasm to facilitate the development of robust genetic markers for ADB^25^. Recently, infrared spectroscopy has been shown to distinguish bark extracts of ash trees with low, intermediate and high susceptibility to ADB^26^. A complementary approach is to identify small molecule chemical markers. These may better reflect the susceptibility to ADB of a given genotype in the context of other biotic and abiotic factors in the location where the tree is growing and provide some mechanistic insight into disease and defence mechanisms of ADB. Untargeted metabolite screening of Danish ash leaf extracts identified five secoiridoid glycoside-like metabolites—P2/N2, P5, P1/N4, P7, N5, and N3, and the related metabolites P3/P4—that were more abundant in ADB susceptible ash trees^27^, leading to the hypothesis that if future breeding strategies selected ADB tolerant trees with reduced iridoid glycosides levels, then there may be inadvertent selection for trees with greater susceptibility to herbivore pests such as EAB.

To extend this study, we undertook a detailed investigation into the structural diversity of secoiridoid glycosides in UK *F. excelsior* leaf extracts from trees predicted to be tolerant and susceptible to ADB. Additionally, we compared secoiridoid glycoside profiles of UK and Danish ash to ascertain whether increased secoiridoid glycoside abundance is also observed in UK ash susceptible to ADB. We also profiled *F. mandschurica* secoiridoid glycosides, as both *A. planipennis* and *H. fraxineus* naturally co-exist on this species^23,28,29^.

## Results

### Identification of previously reported secoiridoid-like compounds in Danish ash

We first examined whether ADB discriminatory secoiridoid glycosides identified in the study of Danish ash were also (i) present in UK ash leaf samples, and (ii) more abundant in susceptible UK ash. We profiled UK samples from polytunnel grown grafts of trees identified in their natural environment, primarily Norfolk, as healthy trees amongst heavily infected and dying trees. Leaves were harvested in late July, similar to the previously sampled Danish trees. The original Danish and new UK leaf material was extracted in parallel (see Methods) and LC–MS/MS data acquired in positive and negative ion modes.

Extracted ion chromatograms were generated corresponding to the compounds **1**–**4** [where **1**(P2/N2), **2**(P5), **3**(P1/N4) and **4**(P7) relate to previous nomenclature putatively assigned to be iridoid glycosides]^27^. **1**–**4** were confirmed in positive and negative ion modes (Fig. 1 and Supplementary Fig. S1–S6). Compound **5** (N5 in Sollars et al.) was only observed in negative ion mode and is an adduct of **1**(N2) having lost a hexose moiety from the [2M-H]^−^ dimer formed in the electrospray source, Supplementary Fig. S4^27,30^. N3 from Sollars et al. was not observed in any of our ash leaf extracts. However, a compound **6**, was observed at the expected retention time for N3 and its peak intensity followed the same trend as observed for N3 with greater abundance observed in susceptible than tolerant Danish leaf extracts (Supplementary Fig. S5). The calculated molecular formula of **6**, C_18_H_25_O_13_ could correspond to [N3-hydroxytyrosol]. We did not observe *m/z* 247.06 (C_11_H_12_O_5_Na) corresponding to the secoiridoid-like metabolites P3 and P4. However, peaks with *m/z* 225.0762 corresponding to [M+H]^+^ adducts (C_11_H_13_O_5_) were observed co-eluting with **1** and **3** (Supplementary Fig. S6), suggesting P3 and P4 may arise from in-source fragmentation of **1** and **3**, respectively. The presence of **1**–**4** and **6** were confirmed in UK ash (Fig. 1, Supplementary S1, S5).

### Identification of characteristic features in mass spectra of secoiridoid glycoside standards

**Figure 2:**
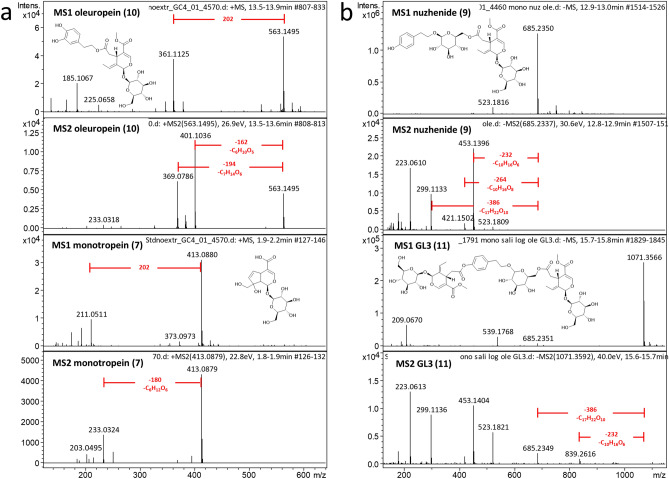
Mass spectra of iridoid glycoside standards in positive ion mode (**a**) and negative ion mode (**b**). (**a**) The predominant peaks arise from [M+Na]^+^ and major fragment ions arise from losses of C_6_H_10_O_5_ (162 Da) and C_7_H_14_O_6_ (194 Da). (**b**) The predominant pseudomolecular ion peak corresponds to [M–H]^−^ and major fragment ions arise from a C_11_H_11_O_5_ (*m/z* 223) fragment and neutral losses of C_10_H_16_O_6_ (232 Da) and C_17_H_22_O_10_ (386 Da).

To identify iridoid related leaf metabolites we first analysed commercial standards of iridoid glycosides, monotropein **7** and loganic acid **8**, and secoiridoid glycosides nuzhenide **9**, oleuropein **10**, and GL3 **11** by LC-HRMS/MS (Fig. 2 and Supplementary Fig. S7–S15). The predominant fragment ions in positive ion mode for secoiridoids **9**–**11** represented losses of 162 Da (C_6_H_10_O_5_) corresponding to loss of a glucosyl moiety and 194 Da (C_7_H_14_O_6_), corresponding to loss of a glucosyl and a methoxy group (Supplementary Fig. S7). MS2 spectra of monotropein **7** and loganic acid **8** feature a predominant peak corresponding to loss of 162 Da (C_6_H_10_O_5_). However, instead of corresponding to loss of 194 Da, the predominant peak corresponds to loss of 180 Da (C_6_H_12_O_6_), indicative of a glucosyl group and H_2_O, implying a hydroxyl group. Minor peaks corresponding to [M–C_6_H_12_O_6_+H]^+^ were also observed in the MS2 spectra of the secoiridoids **9**–**11** (Fig. 2 and Supplementary Fig. S7).

Full scan MS1 spectra for each compound **7**–**11** revealed two discrete peaks with *m/z* difference of 202—the [M+Na]^+^ adduct of each molecule, and a second peak [M–C_6_H_12_O_6_+H]^+^ arising from the in-source fragmentation^31^ and GL3(**11**) showed an additional peak at 713.2427, corresponding to [M–2C_6_H_12_O_6_+H]^+^. **9** and **10** showed the same peak at *m/z* 225.0658 as noted for **1**–**4** (Supplementary Fig.S7). In addition, the MS2 spectrum of oleuropein(**10**) has a minor fragment ion *m/z* = 265.0682 (formula C_11_H_14_O_6_Na), probably representing the secoiridoid aglycone core fragment.

In negative ion mode, MS1 spectra of the standards exhibit one predominant peak corresponding to [M–H]^−^. Fragment ions corresponding to loss of 232 Da (C_10_H_16_O_6_ arising from loss of the glucosyl moiety and rearrangement of the pyran ring)^32,33^ were observed in the [M–H]^−^ MS2 spectra of secoiridoids **9**–**11** but not the C-10 iridoids **7** and **8** (Fig. 2 and Supplementary Fig. S8–S13). MS2 spectra of **7** and **8** exhibit loss of 224—C_7_H_12_O_8_—representing glucosyl, CO_2_ and OH groups (Fig. S9-10)^34^. Additional peaks in the MS2 spectra of nuzhenide(**9**) and GL3(**11**) *m/z* = 299.1131 and *m/z* = 685.2350, respectively, correspond to neutral loss of a 386 Da oleoside-11-methyl ester moiety, C_17_H_22_O_10_^32,35^. *m/z* = 299.1131 corresponds to a salidroside fragment C_14_H_19_O_7_, also observed in the MS2 spectrum of GL3(**11**) (Fig. 2). Minor peaks in the MS2 spectra of nuzhenide(**9**) and oleuropein(**10**) (*m/z* = 421.1502 and 275.0929 respectively) correspond to loss of C_10_H_16_O_8_ (264 Da). Supplementary Figures S9–S13 show assignments of fragment ions from the standards analysed. Additionally, MS2 spectra of the secoiridoids **9**–**11** but not the C-10 iridoids **7** and **8** featured *m/z* 223.0610, (C_11_H_11_O_5_) corresponding to the core secoiridoid aglycone moiety (Fig. 2 and Supplementary Fig. S9–S13). In negative ion mode, loganic acid eluted much earlier due to the eluent used for negative ion mode analyses (Supplementary Fig. S14).

### Confirmation of 1–4 as secoiridoid glycosides and identification of additional secoiridoid glycosides in ash leaves

**Figure 3:**
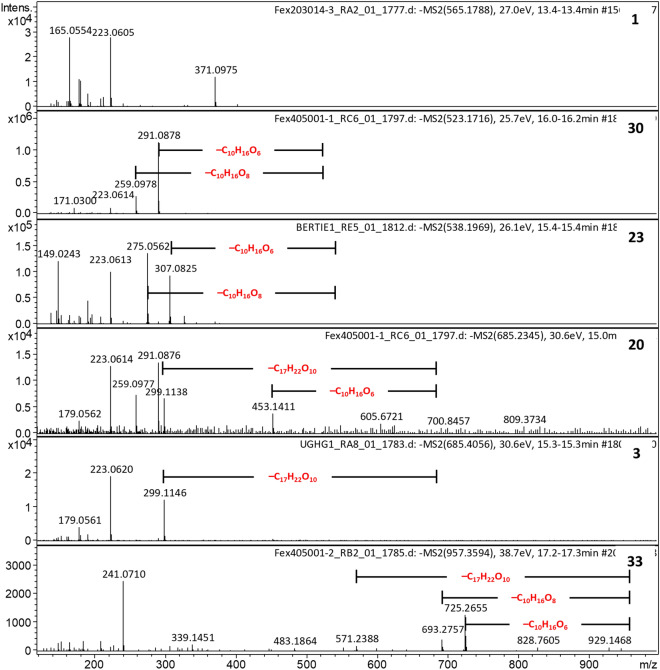
Example MS2 spectra of secoiridoid glycosides in ash leaf extracts in negative ion mode highlighting the characteristic losses of 232 Da (C_10_H_16_O_6_), 264 Da (C_10_H_16_O_8_) and 386 Da (C_17_H_22_O_10_).

Using the above criteria, constant neutral loss chromatograms were generated for 162 Da in positive ion mode and 232 Da and 386 Da losses in negative ion mode for both Danish and UK ash leaf extracts. MS and MS2 spectra for compounds **1**–**4** were compared to the MS2 spectra of the standards, confirming **1**–**4** to be secoiridoids and leading to identification of 22 additional putative secoiridoid glycosides **12**–**33** (examples shown in Fig. 3 and Supplementary Fig. S15).

These findings are summarised in Table 1 and Supplementary Tables S1–S3. Fragment ions and neutral losses from the [M+Na]^+^ and [M–H]^−^ ions for each compound **1**–**33** are visualised in network diagrams (Supplementary Fig. S16–S18 and Supplementary Tables S4 and S5). **6** (related to N3 from Sollars et al.) does have some shared fragments with some of the compounds **12**–**33** assigned as secoiridoids (Supplementary Fig. S17 and Table S5) however it lacks any of the characteristic neutral losses of 232, 264 or 386 observed for secoiridoids.

**Table 1:** Compounds identified as secoiridoid glycosides with key positive and negative mode neutral losses and putative assignments included.

Compound	(pos*) Rt/min	[M+Na]^+^ *m/z*	[M-H]^−^ *m/z*	Molecular formula	Fragments of [M + Na]^+^		Fragments of [M–H]^−^			Assignment	References
					– 162	–194	–232	–264	–386		
*Iridoid glycoside standards*											
**7**	1.2	413.1057	389.1089	C_16_H_22_O_11_	Y	–	–	–	–	Monotropein standard	
8*	12.6	399.1256	375.1297	C_16_H_24_O_10_	Y	–	–	–	–	Loganic acid standard	
**9**	14.9	709.2293	685.2349	C_31_H_42_O_17_	Y	Y	Y	Y	Y	Nuzhenide standard	
**10**	15.7	563.1715	539.1770	C_25_H_32_O_13_	Y	Y	Y	Y	–	Oleuropein standard	
**11**	15.8	1095.3500	1071.3562	C_48_H_64_O_27_	Y	Y	Y	–	Y	GL3 standard	
*Putative iridoid glycosides in ash leaf extracts*											
**12**	12.0	443.1157	not obs	C_17_H_24_O_12_	Y	Y	n/a	n/a	n/a	10-hydroxyoleoside 11-methyl ester	^37^
13*	12.4	427.1213	403.1246	C_17_H_24_O_11_	Y	Y	–	–	–	Oleoside methyl ester/secologanoside methyl ester	^7,38^
**14**	12.7	not obs	525.1606	C_24_H_30_O_13_	n/a	n/a	–	–	–	Demethyloleuropein	^35,39^
**15**	13.1	589.1729	565.1774	C_23_H_34_O_16_	Y	Y	–	–	Y	Methylglucooleoside or isomer	^7^
**16**	13.3	not obs	509.1658	C_24_H_30_O_12_	n/a	n/a	Y	–	–	Demethylligustroside	^39^
**6**	13.3	not obs	449.1301	C_18_H_26_O_13_	n/a	n/a	–	–	–	N3-hydroxytyrosol	^36^
**1**	13.5	589.1728	565.1774	C_23_H_34_O_16_	Y	Y^	–	–	Y	P2/N2 methylglucooleoside or isomer	^7^
**17**	14.8	579.1680	555.1719	C_25_H_32_O_14_	Y	Y	–	–	–	10-hydroxyoleuropein	^36^
**18**	14.9	709.2311	685.2349	C_31_H_42_O_17_	Y	Y	Y	Y^	–	Isomer of nuzhenide/excelside B	^35^
**19**	14.9	441.1376	417.1402	C_18_H_26_O_11_	Y	Y	Y	Y	–	Oleoside dimethyl ester	^7,40^
**20**	15.0	709.2323	685.2349	C_31_H_42_O_17_	Y	Y	Y	Y^	Y	Isomer of nuzhenide/excelside B	^35^
**2**	15.1	709.2311	685.2349	C_31_H_42_O_17_	Y	Y	Y	Y^	Y	Isomer of nuzhenide/excelside B	^27,35^
**21**	15.3	563.1729	539.1170	C_25_H_32_O_13_	Y	Y	–	Y	–	10-hydroxyligustroside	^7^
**3**	15.4	709.2308	685.2349	C_31_H_42_O_17_	Y	Y	Y^	–	Y	P1/N4; isomer of nuzhenide/excelside B	^27^
**22**	15.5	1033.3161	1009.3194	C_46_H_58_O_25_	Y	Y	Y	Y	Y	Oleoacetoside	^35,40–42^
**23**	15.7	563.1725	539.1170	C_25_H_32_O_13_	Y	Y	Y	Y	Y	Oleuropein (**10**)	^7^
**24**	15.9	547.1782	523.1821	C_25_H_32_O_12_	Y	Y	–	–	Y	Excelsioside	^7^
**25**	16.0	741.2935	717.2975	C_33_H_50_O_17_	Y	Y	Y	Y	Y	Isomer of jashemsloside C/D	^43^
26*	16.1	625.2092	601.2138	C_27_H_38_O_15_	Y	Y	–	–	–	Frameroside/2″-epi-frameroside	^44^
**27**	16.2	783.3401	759.3445	C_36_H_56_O_17_	Y	Y	Y	Y	–	Isomer of (9) from *R. glutinosa*	^45^
**28**	16.2	757.2878	733.2924	C_33_H_50_O_18_	Y	Y^	–	–	Y	Jaspofoliamoside A or isomer	^41^
**29**	16.3	933.2989	909.3034	C_42_H_54_O_22_	Y	Y	–	–	–	GL5 or isomer	^7,8,35^
**30**	16.3	547.1783	523.1821	C_25_H_32_O_12_	Y	Y	Y	Y	–	Ligustroside	^7^
**31**	16.7	933.3004	909.3034	C_42_H_54_O_22_	Y	Y	Y	–	Y	GL5 or isomer	^7,8,35^
**4**	17.0	933.2987	909.3034	C_42_H_54_O_22_	Y	Y	–	–	–	P7—GL5 or isomer	^27,35^
32*	17.1	967.3426	943.3453	C_43_H_60_O_23_	Y	Y	–	–	Y	Isomer of pulosarioside	^46^
**33**	17.4	981.3565	957.3609	C_44_H_62_O_23_	Y	Y	Y	Y	Y	Jaspofoliamoside E or isomer	^47^

*indicates compounds with significant retention time shifts (> 2 min earlier) in negative mode compared to positive ion mode due to different mobile phases.

^denotes this peak is observed but is very small; < 3% intensity of base peak.

Of the 26 compounds **1**–**4** and **12**–**33** identified in the *F. excelsior* leaf extracts, 24 were observed in positive ion mode and 25 in negative ion mode. In positive ion mode, 22 compounds share a pair of fragment ions corresponding to neutral losses of 162 (C_6_H_10_O_5_; red edges in Supplementary Fig. S16) and 194 (C_7_H_14_O_6_; black edges in Supplementary Fig. S16) characteristic of the secoiridoid glycoside standards. The predominant pair of fragment ions in the MS2 spectra of **1** and **28**, correspond to [M–2xC_6_H_10_O_5_+Na]^+^ and [M–C_6_H_10_O_5_–C_7_H_14_O_6_+Na]^+^, implying the loss of two glucosyl moieties (Supplementary Fig. S15). As seen in Supplementary Fig. S16, 9 compounds share a fragment ion *m/z* 265.0682 (C_11_H_14_O_6_Na), that was also observed in the oleuropein standard(**10**). Of these, 8 also have the fragment ion *m/z* = 233.0417 (C_10_H_10_O_5_Na). MS2 spectra of [M+Na]^+^ adducts of 10 compounds also have a peak at *m/z* 165.0550 (C_9_H_9_O_3_), possibly representing a fragment derived from a rearranged secoiridoid (Supplementary Fig. S16)^36^.

**Figure 4:**
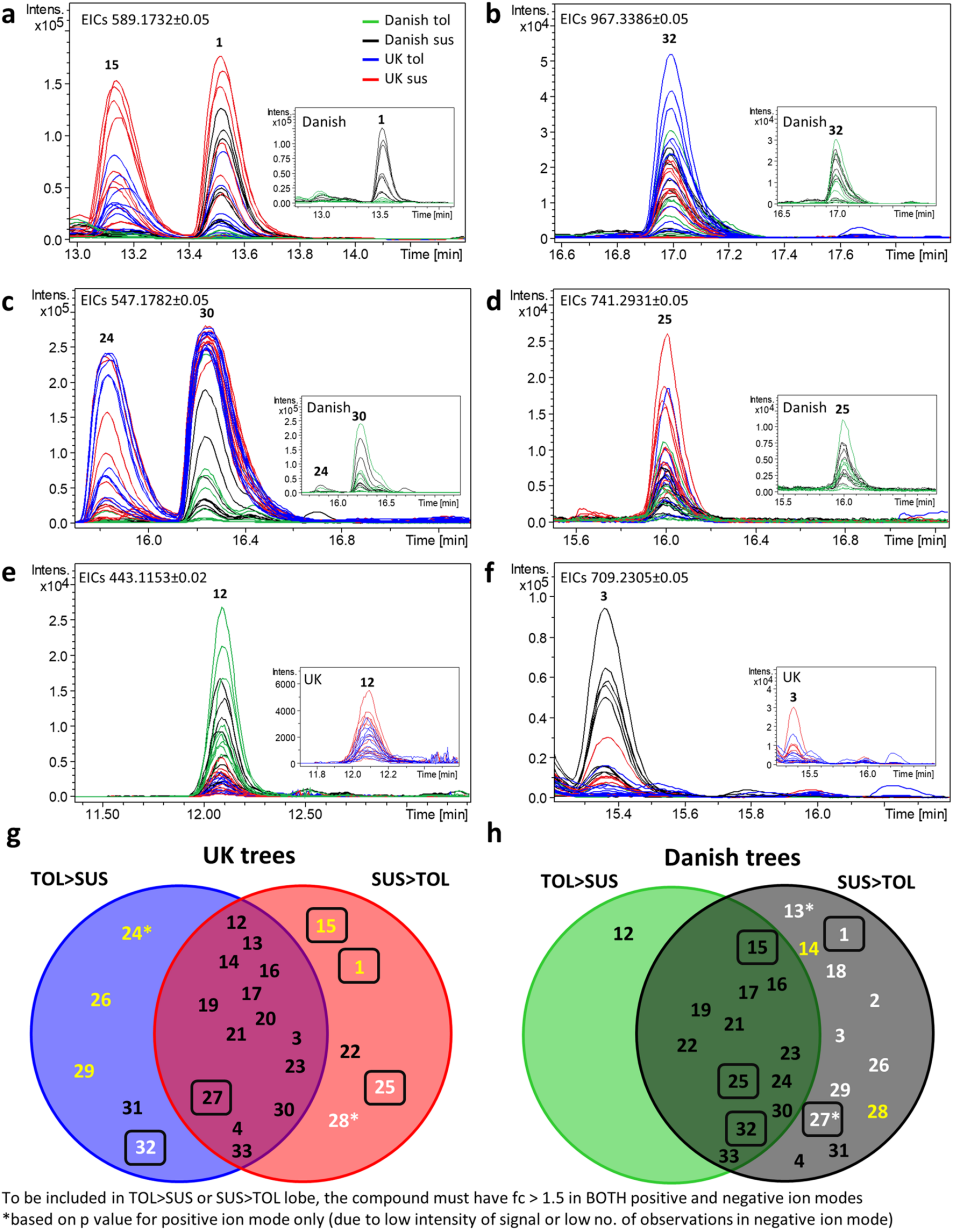
(**a**–**f**) Extracted ion chromatograms showing relative abundance of [M+Na]^+^ adducts of several iridoid glycosides identified in UK and Danish ash leaf extracts. Insets show low intensity EICs only for clarity. Venn diagrams showing relative distribution (fc threshold 1.5) of each compound between tolerant and susceptible genotypes of (**g**) UK trees and (**h**) Danish trees. Compounds shown in white have p value < 0.05 and compounds in yellow have p values < 0.1. **18** and **2** were only observed in 5/24 UK leaf extracts so are omitted from panel (**g**) and **20** was only observed in 7/18 Danish leaf extracts so is omitted from panel (**h**). Compounds proposed to be novel are boxed. **1**–**4** are P2/N2, P5, P1/N4 and P7, respectively from Sollars et al.^27^.

In negative ion mode, 13 compounds displayed fragment ions arising from neutral loss of 232 Da i.e.[M–C_10_H_16_O_6_–H]^−^, indicative of loss of a glucose moiety and rearrangement of the pyran ring characteristic of secoiridoids standards **9**–**11**. Eleven of these compounds also exhibited [M–C_10_H_16_O_8_–H]^−^ fragments corresponding to loss of 264 Da, as in nuzhenide(**9**) and oleuropein(**10**), whereas two compounds, **17** and **21,** instead lose 248 Da (C_10_H_16_O_7_). Crucially, thirteen compounds lose a neutral 386 Da (C_17_H_22_O_10_) fragment, as observed in the MS2 spectra of nuzhenide(**9**) and GL3(**11**), indicative of a terminal oleoside-methyl ester moiety^35^ and eighteen compounds share fragment ion *m/z* 223.0610 (C_11_H_11_O_5_), corresponding to the secoiridoid aglycone core (Supplementary Fig. S17–S18 and Table S5).

Supplementary Figure S16 demonstrates the high degree of similarity between the compounds identified as secoiridoid glycosides, with isomers clustering together, for example **1** and **15** (C_23_H_34_O_16_); **21** and **23** (C_25_H_32_O_13_); **24** and **30** (C_25_H_32_O_12_); **2**, **3**, **18** and **20** (C_31_H_42_O_17_); and **4**, **29** and **31** (C_42_H_54_O_22_). The MS2 spectra of both **1** and **15** suggest loss of 2 glucosyl units therefore** 1** or **15** could be methlyglucooleoside (7-*β*-1-D-glucopyranosyl-11-methyl oleoside), previously isolated from *F. excelsior* leaves, or an isomer thereof^7,35,38^. Thus, if one structure is methlyglucooleoside, the other is novel. In positive ion mode, compounds **21** and **23** (C_25_H_32_O_13_) share fragment ions *m/z* 401, 385, 383, 369 and compounds **24** and **30** (C_25_H_32_O_12_) share a set of fragments *m/z* 385, 369, 367, 353, and 311. Compound **4** (P7; C_42_H_54_O_22_) shares fragments *m/z* 577, 609 and 753 with its isomer **31** in addition to the ion pair *m/z* 771 and 739 that are also present in the MS2 spectrum of the isomer **29**.

In negative ion mode (Supplementary Fig. S17–S18), different fragmentation patterns of these isomers allows some structural inference. For example, compounds **21** and **23** have the same molecular formula as oleuropein, C_25_H_32_O_13_. **23** elutes slightly later, but with identical retention time and fragmentation patterns in positive and negative mode as the oleuropein standard (**10**), thus is assigned as oleuropein. Conversely, the fragmentation patterns of [M+Na]^+^ and [M–H]^−^ adducts of **21** imply a core secoiridoid aglycone with one extra oxygen atom, i.e. *m/z* = 281 (C_11_H_14_O_7_Na) in positive and *m/z* 239 (C_11_H_11_O_6_) in negative ion modes (Supplementary Figs. S16–S19). This could be 10-hydroxyligustroside, previously identified in *F. excelsior* leaves^7,36^. The peak at *m/z* = 281 (C_11_H_14_O_7_Na) in positive ion mode is also present in the MS2 spectra of **12** (C_17_H_24_O_12_Na; assigned as 10-hydroxyoleoside methyl ester) and **17** (C_25_H_32_O_14_; assigned as 10-hydroxyoleuropein)^36,38^, which also has a peak at *m/z* 239 (C_11_H_11_O_6_) in negative ion mode.

Of the two isomers **24** and **30**, (C_25_H_32_O_12_) **30** elutes later and has a pair of fragment ions *m/z* 259.0986 (C_15_H_15_O_4_) and *m/z* 291.0886 (C_15_H_15_O_6_) in negative ion mode, consistent with a secoiridoid aglycone containing a tyrosol moiety as in ligustroside, rather than hydroxytyrosol as in oleuropein (Supplementary Fig. S17, S19), thus is assigned as ligustroside. Conversely, **24**, eluting earlier, has a different fragmentation pattern (Supplementary Figs. S17, S19) and is predicted to be excelsioside, where the iridoid C-7 is linked to the hydroxytyrosol moiety via the phenol O rather than the ethanolic O atom^35,36^. Both have previously been identified in *F. excelsior* leaves^7^.

The *m/z* 259/291 fragment ion pair appears in MS2 spectra of five other compounds, including **4** (P7 from Sollars et al.; C_42_H_54_O_22_) and its isomer **31.** These are absent from the MS2 spectrum of the other isomer **29**. **29** has a number of neutral losses (524 Da, 686 Da, 730 Da, 748 Da) shared with other compounds, (Supplementary Figs. S18, S20). These could be GL5, previously been reported from *F. excelsior*^27,35^, or isomers jaspolyanoside, austrosomide and/or 6′-elenolylnicotiflorine^41,48,49^. The *m/z* 259/291 fragment ion pair is also observed in the MS2 spectra of the nuzhenide isomers **2**, **18** and **20**, and are absent from their isomer **3**. Both **2** and **3** have an additional peak *m/z* 299.1139, suggesting a salidroside moiety, also present in the nuzhenide standard (Fig. 3 and Supplementary Fig. S21).

Three secoiridoids (**13**, **26** and **32**) eluted earlier in negative mode compared to positive ion mode, as did loganic acid(**8**), probably due to the presence of free carboxylic acid moieties in these compounds. Free COOH groups for **26** (C_27_H_38_O_15_) are consistent with frameroside/*epi*-frameroside isolated previously from *F. americana*^44^. **13** and **26** both have fragment ions with *m/z* differences of 44 Da (CO_2_) in their MS2 spectra and **32** loses 44 Da in negative ion mode. **32** also has a peak at *m/z* 667.2617 [M–H]^−^ in its MS2 spectrum corresponding to neutral loss of 276 Da (C_10_H_16_O_6_ + CO_2_; Supplementary Figs. S17–S18, S22), as do **14** and **16** (assigned as demethyloleuropein and demethylligustroside, respectively). As the only known iridoid glycoside with the same molecular formula C_43_H_60_O_23_ as **32** is the secoiridoid pulosarioside, isolated from *Alyxia reinwardtii*^46^, which lacks a terminal carboxylic acid group, we predict **32** is a novel compound.

Of the remaining compounds, **28** and **33** have not previously been reported from *F. excelsior* and are assigned as jaspofoliamosides A and E, respectively, or isomers thereof^41,47^. **22** has the same formula as secoiridoids oleoacetoside and isooleoacetoside (C_46_H_58_O_25_)^40,42^, though only oleoacetoside has previously been reported from *F. excelsior*^35^ therefore we assign **22** as oleoacetoside. Several others (**25**, **27**, **32**) probably represent new compounds. The only known iridoid glycosides with the same molecular formula as **25** (C_33_H_50_O_17_) are jashemslosides C and D, from *Jasminum hemsleyi* though these are not secoiridoids^43^. However, characteristic neutral losses of 232 Da, 264 Da and 386 Da in the [M–H]^−^ MS2 spectrum of **25** and losses of 162 Da and 194 Da in its [M+Na]^+^ MS2 spectrum all suggest **25** is a secoiridoid (Supplementary Figs. S16–S18, S23, Table 1). The molecular formula of **27** (C_36_H_56_O_17_) is consistent with an unnamed C-10 iridoid isolated from *Rehmannia glutinosa*^45^, although our data clearly indicate neutral losses of 162 Da and 194 Da in positive ion mode and neutral losses of 232 Da and 264 Da in negative ion mode, (Table 1, Supplementary Figs. S16–S18, S24) indicative of a secoiridoid. Therefore we predict that **27** is another new secoiridoid.

### Distribution of secoiridoid glycoside metabolites in UK and Danish tolerant and susceptible *F. excelsior* leaf extracts and Manchurian ash

**Figure 5:**
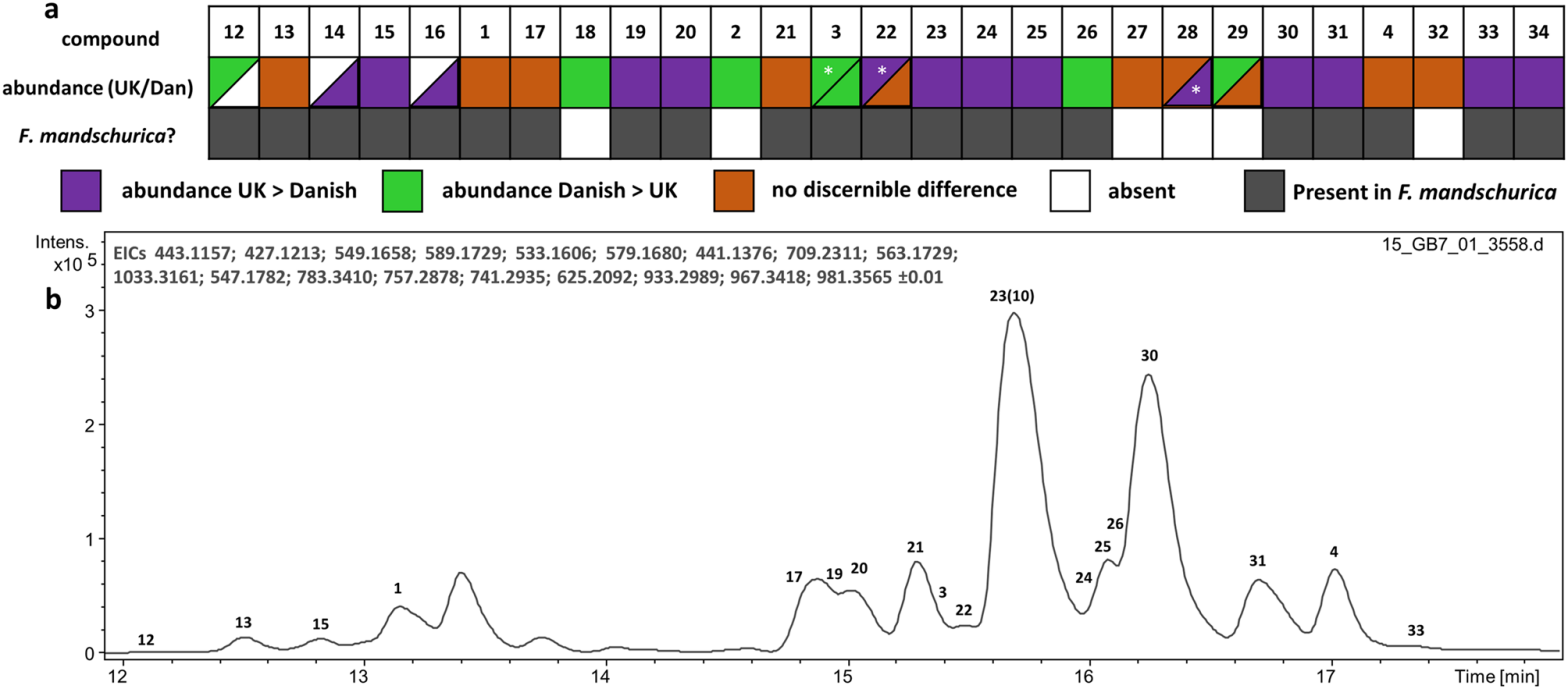
(**a**) Heat map showing relative abundance of **1**–**34** in UK and Danish *F. excelsior* (top row) and presence/absence of **1**–**34** in *F. mandschurica*. Green and purple shading indicate the difference between peak areas of each compound in UK and Danish samples that have p value < 0.05. *p value < 0.1. Cells that are split indicate that there was a different outcome of the t-test for [M+Na]^+^ and [M–H]^−^ adducts of these compounds. (**b**) Extracted ion chromatograms representing [M+Na]^+^ adducts of **1**–**33** observed in *F. mandschurica*.

The relative distributions of each compound between tolerant and susceptible UK and Danish ash samples were assessed by overlaying their extracted ion chromatograms and by fold change and t test on normalised peak areas, as illustrated in Figs. 4 and 5 and Supplementary Table S6–S8. Compound **1** was more abundant in susceptible UK ash leaf extracts compared to tolerant leaf extracts (t = − 1.8511, p 0.0776, d.f = 22), (Fig. 4a), though the different abundance of **1** between the Danish susceptible and Danish tolerant groups were more pronounced (t = − 3.7532, p 0.00174, d.f = 16). **28** discriminates susceptible from tolerant UK leaf extracts sampled (t = − 2.1913, p 0.0393, d.f = 22) and Danish leaves sampled (t = − 1.7874, p 0.0928, d.f = 16). **24** and **30** (excelsioside and ligustroside, respectively; Fig. 4c) are more abundant in the UK compared to Danish leaves, with **24** being slightly enriched in the UK tolerant subgroup. Conversely, **12** may discriminate tolerance in Danish leaf extracts (Fig. 4e) whereas **32** discriminates tolerant from susceptible UK leaves sampled but not tolerant Danish leaves sampled (Fig. 4b). **3** (an isomer of nuzhenide; Fig. 4f) is more abundant in the Danish group and enriched in the highly susceptible subgroup, whereas **25** both discriminates susceptibility in UK leaf extracts, and is much more abundant in the UK leaf extracts compared to Danish leaf extracts (Fig. 4d).

The distribution of secoiridoids in susceptible and tolerant groups of UK and Danish trees analysed are illustrated in Fig. 4g and h. These data reveal a dramatic difference in the relative distribution of secoiridoids between the tolerant and susceptible UK and Danish tolerant and susceptible groups. Compounds **1** and **28** appear to discriminate susceptibility in both UK and Danish groups. Fold change analysis showed ten additional compounds **2**, **3**, **4** (original biomarkers of ADB), **13**, **14**, **18**, **26**, **27**, **29** and **31** are more abundant in susceptible than tolerant Danish leaves sampled. However three of those compounds (**26**, **29** and **31**) are more abundant in UK tolerant compared to susceptible trees sampled and the difference in abundance of **31** were not significant (p > 0.1). Two compounds (**2** and **18**) were only detected in 5/24 UK leaf extracts and **13** shows no difference between UK tolerant and susceptible trees sampled (Supplementary Figs. S25–S26). Conversely, of the remaining four compounds that distinguish the UK susceptible group (**15**, **22**, **25** and **28**), low abundance of **15** and **25** in Danish trees precluded discrimination between tolerant and susceptible groups and the greater level of **22** in the UK susceptible group was not statistically significant (p > 0.1) The other two compounds higher in abundance in the UK tolerant group, **24** and **32**, do not clearly discriminate tolerance in the Danish group. Conversely, fold change analysis showed **12** was more abundant in Danish tolerant leaf extracts (Fig. 4e), although this difference was not statistically significant (p = 0.1122).

The differences in distribution of **1**–**4** and **12**–**33** between the UK and Danish populations are summarised in Fig. 4g and h and Fig. 5. In total, 13/26 secoiridoid glycosides were more abundant in UK compared to Danish leaf extracts (fold change > 1.5 and p value < 0.1), including one molecule (**20**) only detected in 7/18 of the Danish samples (Supplementary Fig. S1). In general, the secoiridoids more abundant in UK extracts tended to be larger and more structurally complex, with the exception of **15** (methlyglucooleoside or isomer) and **19** (oleoside dimethyl ester). By contrast, 6/26 secoiridoid glycosides were present in Danish leaf extracts in greater amounts (fold change > 1.5 and p value < 0.1). Two of these compounds, **2** and **18** were only observed in 5/24 of the UK samples, and of the remaining four, one is a smaller secoiridoid **12** (10-hydroxyoleoside 11-methyl ester).

We additionally profiled *Fraxinus mandschurica* leaf extracts by (i) generating extracted ion chromatograms for [M+Na]^+^ and [M–H]^−^ for all secoiridoid glycosides **1**–**33** and (ii) constant neutral loss chromatograms for 162 Da and 194 Da in positive ion mode and 232 Da and 386 Da in negative ion mode. This revealed that 20 out of 26 of secoiridoid glycosides **1**–**33** were observed in *F. mandschurica*, including 12 compounds more abundant in UK than Danish *F. excelsior* extracts and 4 compounds that were more abundant in Danish *F. excelsior* extracts (Fig. 5). We identified an additional secoiridoid glycoside, **34**, that has the same molecular formula as jaspolyoside and its isomers isojaspolyoside A-C^50^. Reanalysing UK and Danish spectra, **34** was detected in UK extracts and lower amounts detected in Danish extracts (Fig. 5 and Supplementary Fig. S27).

## Discussion

This study, for the first time, comprehensively profiled secoiridoid glycosides in a panel of UK ash leaves and compared them to validated ADB susceptible and tolerant Danish leaf extracts. We significantly expand our original identification of four secoiridoid glycosides **1**–**4** enriched in susceptible Danish genotypes of *F. excelsior* to 27 *F. excelsior* secoiridoid glycosides. Seven of these had not been previously reported and at least four (**25**, **27**, **32** and **1** and/or **15**) are predicted to represent new structures^2, 35,39^. Our data reinforce our previous finding that compound **1** is a discriminatory metabolite of ADB susceptibility in both UK and Danish ash trees sampled. Notably, of the new secoiridoid glycosides identified; **15** and **25** are much more abundant in the UK group and, interestingly, discriminate susceptibility in the UK but not the Danish group. For compounds **27** and **32**, there was no difference in abundance between samples when grouped by geographical origin. However, within the Danish sample group, **27** was more abundant in susceptible compared to tolerant leaf extracts, whereas within the UK sample group, **32** was more abundant in the tolerant compared to susceptible leaf extracts. Furthermore, we undertook the most comprehensive profiling of secoiridoids in *F. mandschurica* leaf extracts to date, substantially increasing the number of secoiridoids detected in this species to 21^51–53^.

A key finding is that secoiridoid abundance is generally not correlated with ADB tolerance or susceptibility. Rather, our comprehensive profiling enabled us to identify specific secoiridoid glycosides that may serve as potential biomarkers of ADB tolerance or susceptibility. Of the secoiridoids initially reported as markers of ADB susceptibility in Danish leaf extracts^27^, we confirmed **1** was also present in higher levels in leaf extracts from susceptible compared to tolerant UK trees sampled (Fig. 4), thus representing a possible robust biomarker of ADB susceptibility in European ash. Particularly interesting was the geographical specificity in distribution and abundance of secoiridoid glycosides. For example, compounds **26** and **29** were more abundant in susceptible Danish, yet more abundant in tolerant UK leaf extracts analysed, highlighting heterogeneity in distribution and raising the question of what selective advantage such heterogeneity confers. While a core subset of seven compounds (**16**, **17**, **19**, **21**, **23**, **30**, **33**) did not appear to distinguish ADB tolerance from susceptibility within either the UK or Danish leaves sampled, five of these compounds are more abundant in the UK compared to Danish leaf extracts, reflecting a geographic influence on these secoiridoid levels. Whether these have evolved due to biotic pressures in their respective environments needs further investigation. In summary, our results suggest there is greater abundance of secoiridoid glycosides in UK than Danish *F. excelsior* leaf extracts (Fig. 5), and notably, these are generally more structurally complex. This could arise from altered gene expression, or activity of enzymes utilising different secoiridoid biosynthetic intermediates in UK ash. Given the associated metabolic cost, the reason for this diversity in structure and abundance is of fundamental interest. It will be important to determine whether these differences discussed here are predictive of ADB tolerance and susceptibility in the wider UK population; larger scale sampling is planned to address this.

It is particularly striking that, whilst our study involved a relatively small number of genotypes and grafts of each genotype, both **15** (potentially the same structure as methlyglucooleoside) and **1** (N2) correlated to susceptibility to ADB in our study. This observation is in agreement with a recent study of bark extracts from a larger panel of *F. excelsior*^54^. Other commonalities included a molecule with the same HRMS *m/z* (943.3353) as **32** (although no MS2 data is available to validate this) that was enriched in tolerant ADB genotypes. Thus, **1**, **15** and **32** may be robust markers of relative ADB susceptibility in bark and leaf tissues^54^. As observed in our Danish leaf extracts, **26** (frameroside/*epi*-frameroside) and one compound *m/z* = 909.2920 also had a weak association with ADB susceptibility. While it is tempting to speculate that this molecule has the same structure as **31** or **4**, further chemical interrogation is needed to validate this prediction.

In its native environment, *F. mandschurica* co-exists with both EAB and ADB^28,29,55^, so it is interesting that our panel of European ash leaves showed greater diversity of secoiridoids than *F. mandschurica* with only 21 out of 27 *F. excelsior* secoiridoids identified in *F. mandschurica*. Of the six *F. excelsior* specific secoiridoids, two of them (**2** and **18**) were much more abundant in the Danish leaf extracts. Notably, **28** was enriched in susceptible populations of both UK (p = 0.0393) and Danish trees (p = 0.0928). Interestingly, **1**, **3** and **4**, enriched in susceptible Danish *F. excelsior* genotypes, were all observed in *F. mandschurica* leaf extracts.

In a previous study of Swedish *F. excelsior* seedlings treated with the *H. fraxineus* toxin viridiol, oleuropein **23**(**10**) levels were lower in an ADB tolerant genotype, whereas demethylligustroside **16** and demethyloleuropein **14** accumulated more in another tolerant genotype. However the overall differences in secoiridoid profile between tolerant and susceptible populations were much more ambiguous^39^. In our study of older, unchallenged trees, **16** and **23** did not discriminate tolerant from susceptible genotypes, although **14** was slightly enriched in Danish susceptible trees (Fig. 4; p = 0.0846).

Whilst EAB larvae feed in the phloem, adults feed on ash leaves. If secoiridoids do contribute to EAB tolerance as feeding deterrents, UK trees may exhibit greater tolerance as higher levels of the secoiridoids identified were observed in the UK leaves sampled. While detailed feeding studies are needed to assess this, biological activity may be further complicated by subsequent chemical modification upon herbivory of this diverse repertoire of secoiridoids^16^. Interestingly, the diversity of secoiridoids in *F. excelsior* identified here and in bark extracts is greater than that reported in green and white ash species native to North America^2,44,52,54^. *F. excelsior* may be more tolerant to EAB than *Fraxinus* species native to North America as recently reported^56^, consistent with complementary studies showing that adult *A. planipennis* has a much greater feeding preference for leaves of green, white and black ash compared to Manchurian and European ash^57^. It remains to be determined whether *F. excelsior* in European forests is more or less tolerant to *A. planipennis* than *F. pennsylvanica*^24^. Clearly, establishing any biological function for the diversity of the secoiridoids identified in leaf (this study) and bark extracts of *F. excelsior* on tolerance or susceptibility to EAB requires further analytical dissection. Our study, along with the recent analysis of *F. excelsior* bark secoiridoids^54^ provides the necessary analytical framework to further pursue such studies and represents a complementary approach to the FT-IR based identification of trees tolerant and susceptible to ADB using bark extracts^26^. A future objective will be to increase sampling density and geographic distribution of *F. mandschurica* and *F. excelsior* leaf extracts for secoiridoid profiling.

In conclusion we found an unexpected diversity of secoiridoids in European *F. excelsior* leaf extracts. While secoiridoid glycosides collectively are not suitable biomarkers to distinguish ADB susceptible from tolerant UK ash trees, we identify several specific secoiridoid glycosides enriched in trees sampled from different locations that could be potential markers of ADB susceptibility (**3** and **27** in Danish ash and **25** in UK ash) and tolerance (**32** in UK ash), with **1** and **28** emerging as robust markers of susceptibility in both sample groups. Encouragingly, the diversity and geographic variability of secoiridoid glycosides uncovered in these European *F. excelsior* samples may prove valuable reservoirs of antifeeding deterrents in mitigating the future threat of EAB. We currently lack knowledge of differences in the overall metabolic landscape of ADB tolerant and susceptible genotypes in a comprehensive, representative sample set. To address this, untargeted analysis of UK ash leaf extracts is underway to find further discriminatory metabolites that could be used as biomarkers for ash dieback susceptibility and tolerance among the wider UK population.

## Methods

UK *F. excelsior* samples were grafted trees taken from woodlands that were deemed to be tolerant (four genotypes—BERTIE, CHRISTINA, RABBIT, VITALSTATISTIX), having survived in locales with high levels of disease and are surrounded by trees that are dead or dying from ADB. These individuals were monitored for leaf and bark damage at least twice per year from 2015. As of December 2019, these trees are still alive in their native environments. UK trees classified as susceptible (four genotypes—Fex203014, Fex203015, Fex303003, Fex405001) have succumbed to the disease in their native woodland. Three ramets (clones) of each genotype were sampled. Trees were grafted in February and March 2016 and transferred to a polytunnel. Leaves were harvested from three separate grafts of each genotype in July 2017, snap frozen in liquid nitrogen and lyophilised. Danish leaf samples were prepared from same leaf material used in Sollars et al.^27,30^. Leaf material from 18 month old grafts of three each of the tolerant (HGH-A, HGH-C, HGH-D) and susceptible (UGH-F, UGH-G, UGH-H) genotypes were selected. Manchurian ash samples were prepared from leaf material harvested from grafted *F. mandschurica* trees in 2014 (three samples). Lyophilised leaf material was ground to powder and 10 mg aliquots were extracted on ice in 400 µL 80% methanol containing 2.5 µg/mL d_5_-IAA then once more in 400 µL 80% methanol and supernatants combined, as reported in Sollars et al.^27^.

Samples were submitted to UPLC-HRMS analysis using a Dionex UltiMate 3000 UHPLC system and Agilent Eclipse Plus C18 UPLC column (2.1 × 150 mm, 1.8 µm particle size) with outflow routed to a Bruker MaXis II Q-TOF with an electrospray source. Samples were run in positive ion mode and then in negative ion mode. Solvents A and B were water and acetonitrile, respectively. For positive ion mode analyses, 0.1% formic acid was added to the mobile phase and for negative ion mode analyses, 0.1% ammonia was added. Compounds were eluted using the following gradient: isocratic elution with 95% solvent A and 5% solvent B for 5 min, followed by a gradient elution to 100% solvent B over min 28.7 min. 5 µL sodium formate (10 mM) was loop-injected as an internal standard. Samples were also analysed using the same elution profile in auto MS/MS mode with the three most intense peaks selected for MS2 data acquisition after each full scan. Standards of oleuropein, nuzhenide, GL3 and loganic acid were purchased from Extrasynthese (France) and monotropein was purchased from PhytoLab (Germany). Indole-2,4,5,6,7-d_5_-3-acetic acid (d_5_-IAA) was purchased from Sigma Aldrich (UK). A mixture of 10 µg/mL each of the iridoid glycoside standards was prepared in 80% methanol and submitted to UPLC-MS/MS analysis.

Peak areas for ions of interest were extracted using XCMS^58 ^ and checked by manually picking peaks in Bruker Compass Data Analysis. Misshaped peaks (i.e. for ions with closely eluting isomers or poorly defined peak shapes) were integrated manually using the peak-picking tool in Compass Data Analysis. Statistical analyses were performed using MetaboAnalyst v4.0 ( https://www.metaboanalyst.ca/ ).^59 ^Peak areas were normalised to the Na(NaCOOH)_3_ adduct (*m/z* = 226.9515) of the sodium formate internal standard (positive ion mode) and the HCOO(NaCOOH)_4_ adduct (*m/z* = 316.9479) in negative ion mode. Missing values were replaced with 1/5 intensity of the minimum observed intensity for that compound. For the data in Fig. 4, samples were grouped into ‘tolerant’ and ‘susceptible’ groups and analysed separately based on location (UK and Denmark) and samples were grouped by geographical location for Fig. 5. Peak areas for each compound were submitted to fold change analysis (threshold 1.5) and t-test. Supplementary Tables S6–S8 show fc, t and p values used for generating Fig. 4 and 5.

## Supplementary information

Supplementary information.

## Data Availability

The datasets generated during and analysed during the current study are available from the corresponding author on reasonable request.
