# Supplementary material for: Diversity of secoiridoid glycosides in leaves of UK and Danish ash provide new insight for ash dieback management
Source: Sci Rep. 2020 Nov 11;10:19566. doi: 10.1038/s41598-020-76140-z (PMC7658974; doi:10.1038/s41598-020-76140-z)
Supplement: Supplementary file 1 — Supplementary information. [file 41598_2020_76140_MOESM1_ESM.pdf]

Supporting Information for

Diversity of secoiridoid glycosides in leaves of UK and Danish ash provide new insight for ash dieback management

John D Sidda,[1,2]* Lijiang Song,[2] Jack L. Parker,[1] David J. Studholme,[3] Christine Sambles,[3] Murray Grant[1]*

[1] School of Life Sciences, University of Warwick, Coventry, UK, CV4 7AL

[2] Department of Chemistry, University of Warwick, Coventry, UK, CV4 7AL

[3] School of Biosciences, University of Exeter, Exeter, UK, EX4 4QD

*corresponding authors

Supplementary Figures and Tables

Supplementary Figure S1 – Extracted ion chromatograms for $[M+Na]^+$ adducts of **1-4** in Danish and UK ash.

Supplementary Figure S2 – Extracted ion chromatograms and mass spectra in **1-4** in negative ion mode.

Supplementary Figure S3 – MS2 spectra of **1-4** in positive and negative ion modes.

Supplementary Figure S4 –Extracted ion chromatograms and mass spectrum showing N2(**1**) and N5(**5**).

Supplementary Figure S5 – Extracted ion chromatograms (*m/z* = 449.1299) of compound **6**, in Danish and UK ash.

Supplementary Figure S6 – Extracted ion chromatograms of **1-4** and *m/z* = 225.0762 representing the putative iridoid glycosides P3/P4 from Sollars *et al*.

Supplementary Figure S7 – Positive ion mode MS1 and MS2 spectra of iridoid glycoside standards **7-11**.

Supplementary Figure S8-13 – Negative ion mode MS1 and MS2 spectra of iridoid glycoside standards **7-11**.

Supplementary Figure S14 – Extracted ion chromatograms of **8-10** in positive and negative ion modes.

Supplementary Figure S15 – Example positive ion mode MS2 spectra for $[M+Na]^+$ adducts of secoiridoid glycosides identified in ash leaf extracts.

Supplementary Table S1 and S2 – Positive ion mode MS2 data for **1-33**.

Supplementary Table S3 – Negative ion mode MS2 data for **1-33**.

Supplementary Figure S16 – Molecular network of fragment ions from MS2 spectra of $[M+Na]^+$ of **1-33.**

Supplementary Figure S17 – Molecular network of fragment ions from MS2 spectra of $[M–H]^–$ of **1-33.**

Supplementary Figure S18 – Molecular network of neutral losses from MS2 spectra of $[M–H]^–$ of **1-33.**

Supplementary Table S4 and S5 – Shared fragment ions in MS2 spectra of $[M+Na]^+$ adducts of **1-33** in positive and negative ion modes.

Supplementary Figure S19 – Negative ion mode MS2 spectra of **21**, **23**, **24** and **30** and oleuropein **10**.

Supplementary Figure S20 – Negative ion mode MS2 spectra of **4**(P7) and its isomers **29** and **31**.

Supplementary Figure S21 – Negative ion mode MS2 spectra of nuzhenide(**9**) and isomers **2**(P5), **3**(P1/N4), **18** and **20**.

Supplementary Figure S22 – Extracted ion chromatograms and MS2 spectra of **32**.

Supplementary Figure S23 – Extracted ion chromatograms and MS2 spectra of **25**.

Supplementary Figure S24 – Extracted ion chromatograms and MS2 spectra of **27**.

Supplementary Table S6 – Statistical analyses (fold change and t-test) of secoiridoids in Danish ash leaves associated with tolerance and susceptibility to ash dieback used to generate Figure 4h

Supplementary Table S7 – Statistical analyses (fold change and t-test) of secoiridoids in Danish ash leaves associated with tolerance and susceptibility to ash dieback used to generate Figure 4h

Supplementary Table S8 – Fold change (threshold 1.5) and t test to demonstrate abundance of compounds **1-34** between Danish and UK ash leaf extracts used to generate Figure 5a

Supplementary Figure S25 – Extracted ion chromatograms of compounds **13** and **26**.

Supplementary Figure S26 – Extracted ion chromatograms of **29** and **31**.

Supplementary Figure S27 – Extracted ion chromatograms and MS2 spectra of **34**.

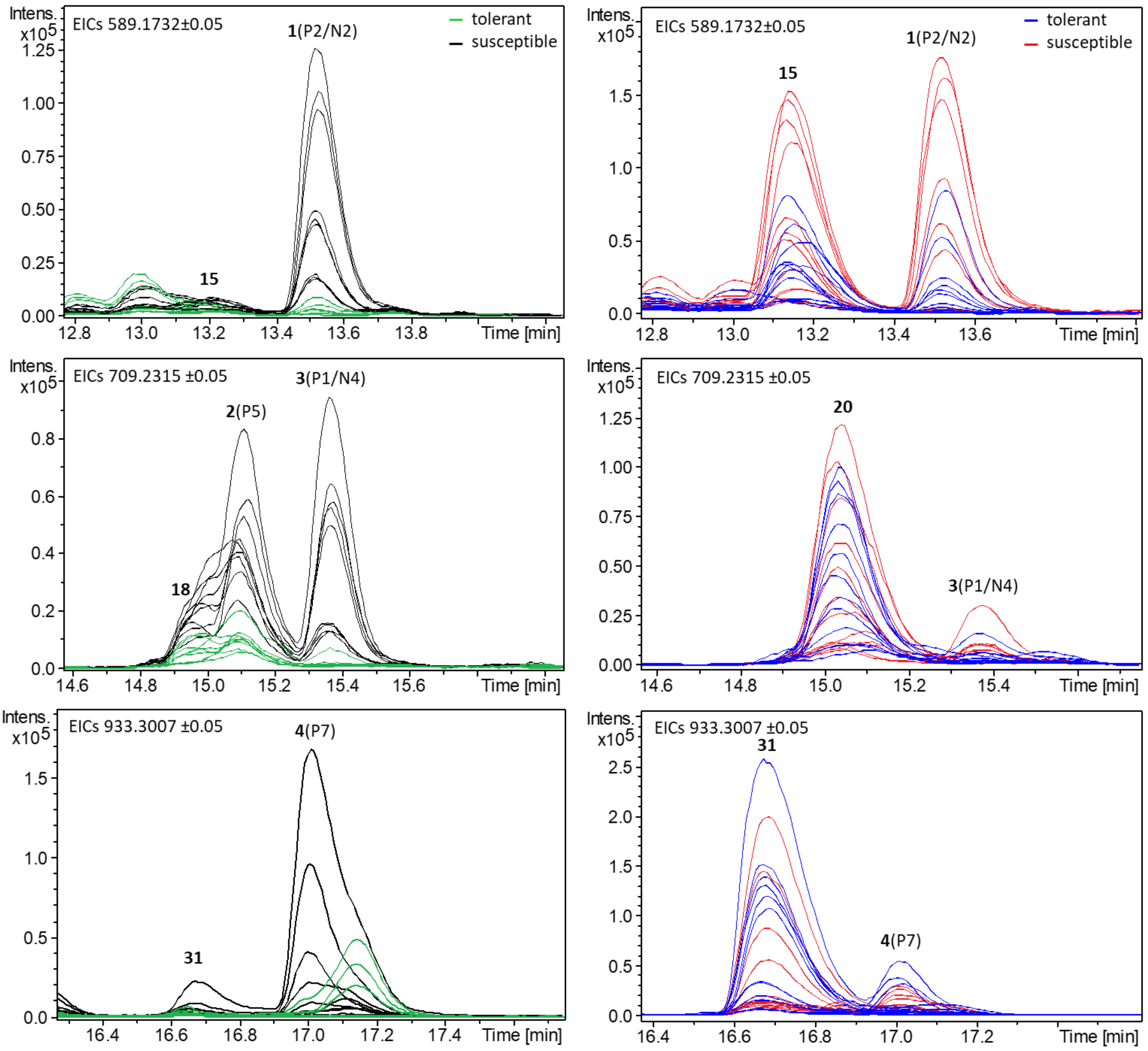

Supplementary Figure S1 – Extracted ion chromatograms for $[M+Na]^+$ adducts of compounds **1**(P2/N2), **2**(P5), **3**(P1/N4) and **4**/P7 putatively identified as secoiridoid glycosides in Sollars *et al* confirming the distribution of these compounds and their isomers in ash leaf extracts of susceptible and resistant Danish trees (left panel) and UK trees (right panel).

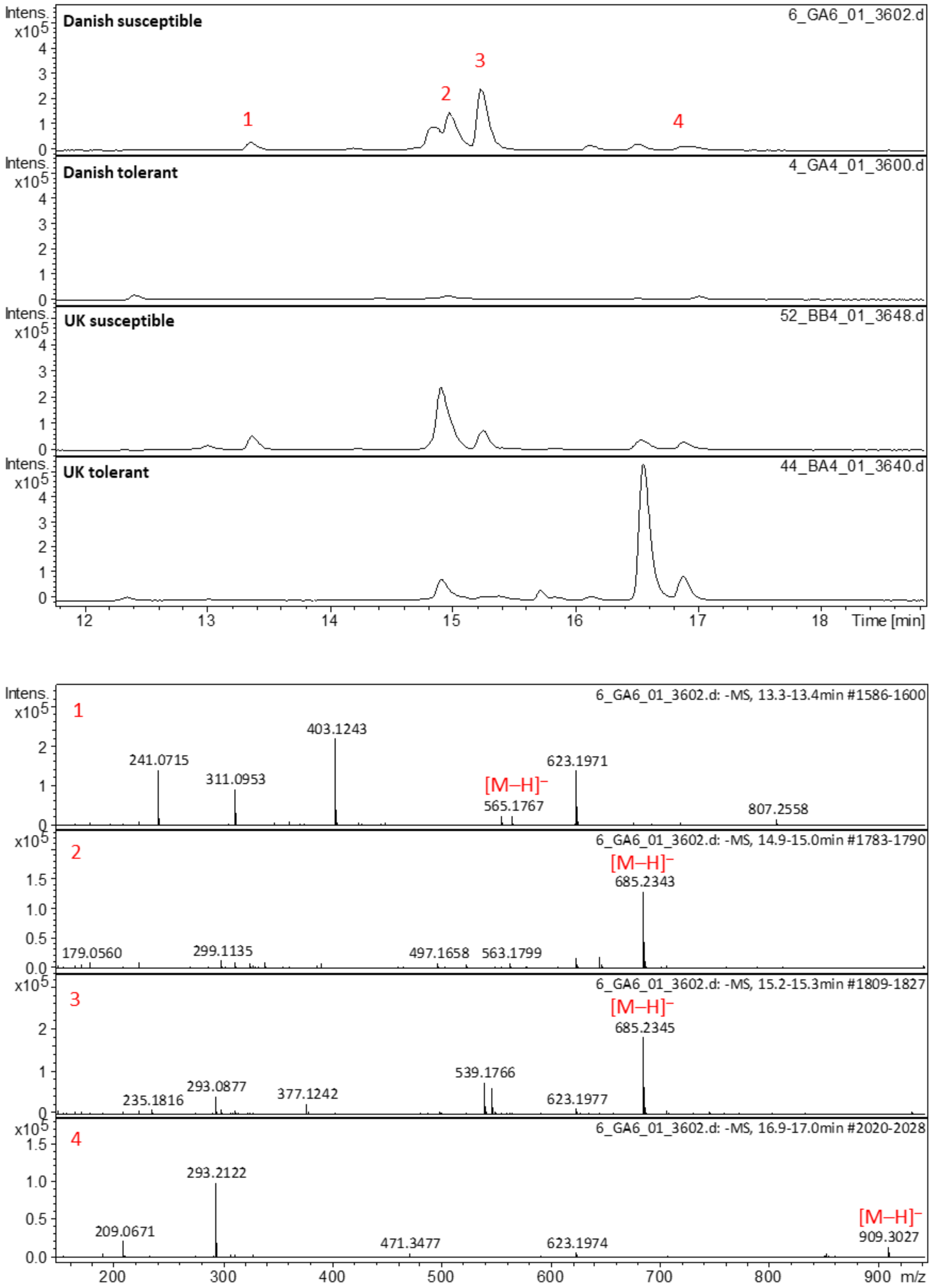

Supplementary Figure S2 – Top panel: Extracted ion chromatograms in negative ion mode corresponding to *m/z* 565.1767 (**1**/P2/N2), 685.2354 (**2**/P5 & **3**/P1/N4) and 909.3024 (**4**/P7); only 1 of each Danish and UK tolerant and susceptible trees shown for clarity. Bottom panel: Negative ion mode mass spectra of **1-4**.

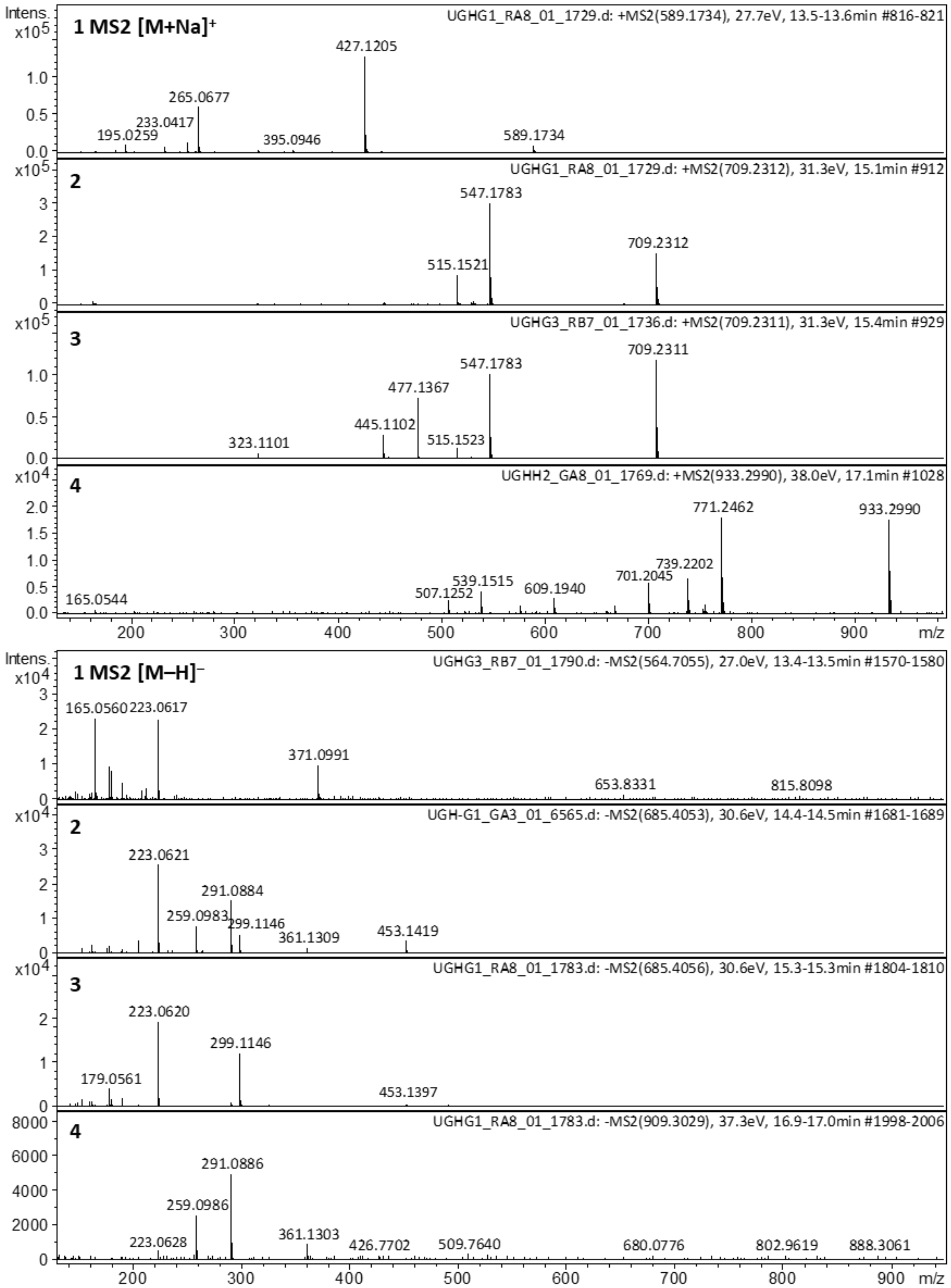

Supplementary Figure S3 – MS2 spectra of **1**(P2/N2), **2**(P5), **3**(P1/N4) and **4**(P7) in positive ion mode (upper panel) and negative ion mode (lower panel).

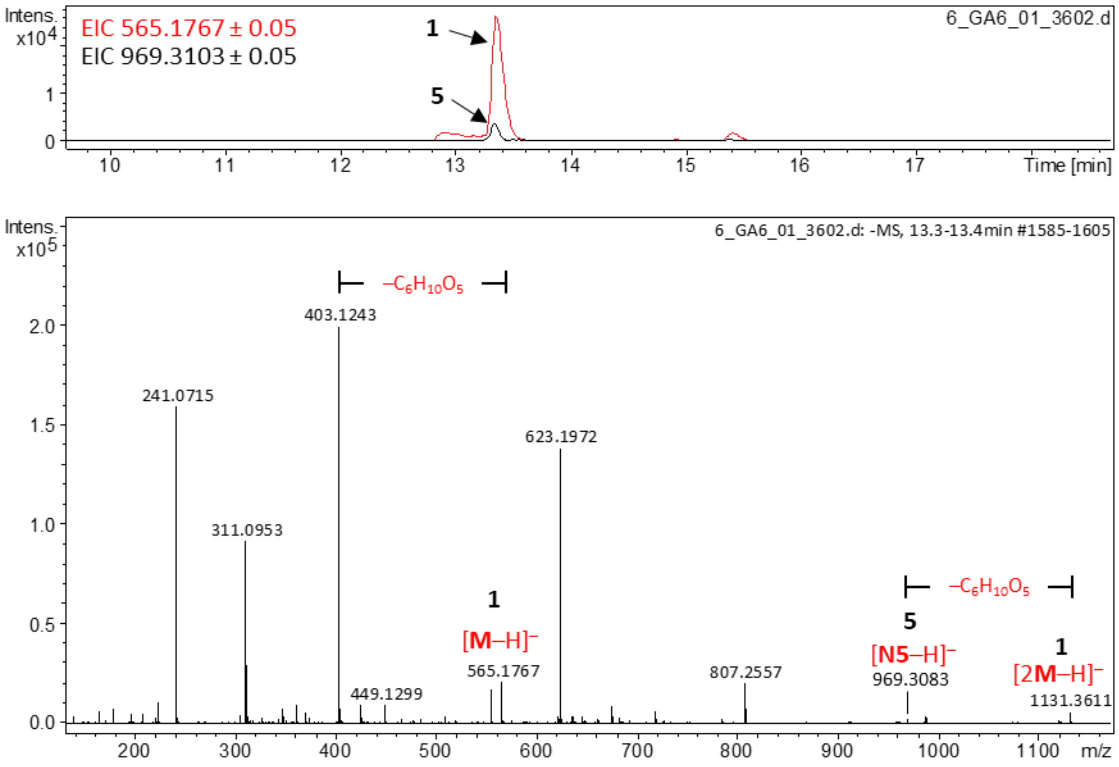

Supplementary Figure S4 – Extracted ion chromatograms *m/z* = 565.1767 (black; **1**) and 969.3081 (red; **5**) representing compounds N2(**1**) and N5(**5**) demonstrating that they co-elute.Mass spectrum of **1**(N2) and **5**(N5) with assignments of adducts of **1**(N2). **5**(N5) is an artefact arising from loss of a hexose moiety from the adduct of the **1** [2M-H]⁻ dimer at *m/z* 1131.3611.

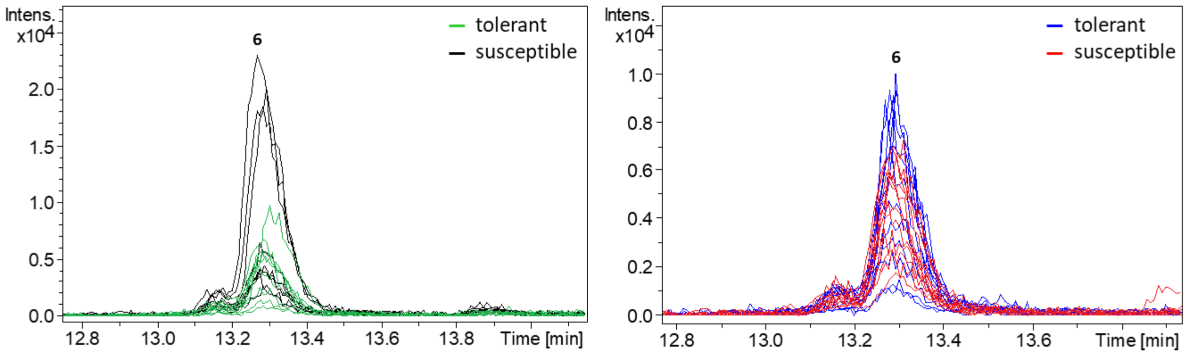

Supplementary Figure S5 – Extracted ion chromatograms (*m/z* = 449.1299) of compound **6**, proposed to be related to N3 from Sollars *et al* in Danish (left) and UK (right) leaf extracts. Compound **6** has molecular formula $C_{18}H_{25}O_{13}$ and possibly corresponds to [N3–hydroxytyrosol–H]⁻.

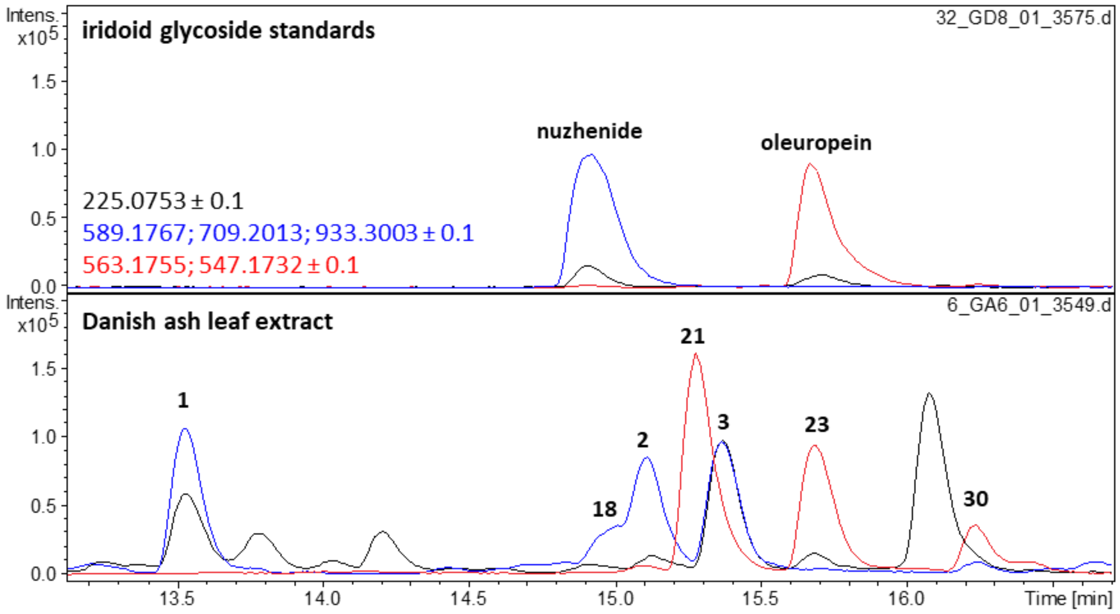

Supplementary Figure S6 – Extracted ion chromatograms *m/z* = 589.1787, 709.2315, 933.3007 of compounds **1**(P2/N2), **2**(P5) & **3**(P1/N4) and **4**(P7) and *m/z* = 225.0762 representing the putative iridoid glycosides P3/P4 from Sollars *et al* in an ADB susceptible Danish tree leaf extract. The black EIC for *m/z* = 225.0762 also overlaps with EIC *m/z* 563.1755, 547.1732 of other known secoiridoid glycosides oleuropein and ligustroside (red).

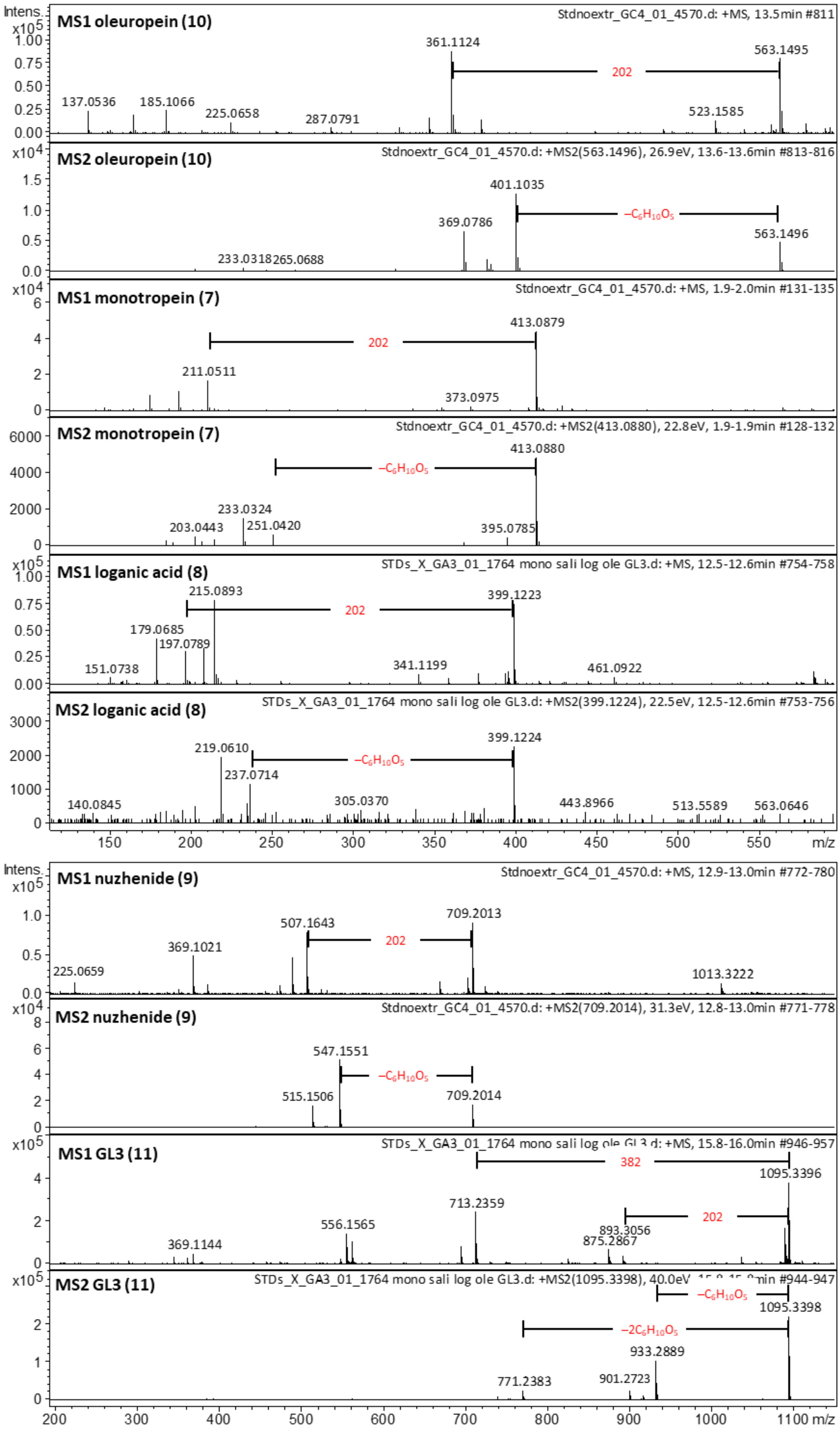

Supplementary Figure S7 – Positive ion mode MS1 and MS2 spectra of iridoid glycoside standards used in this study.

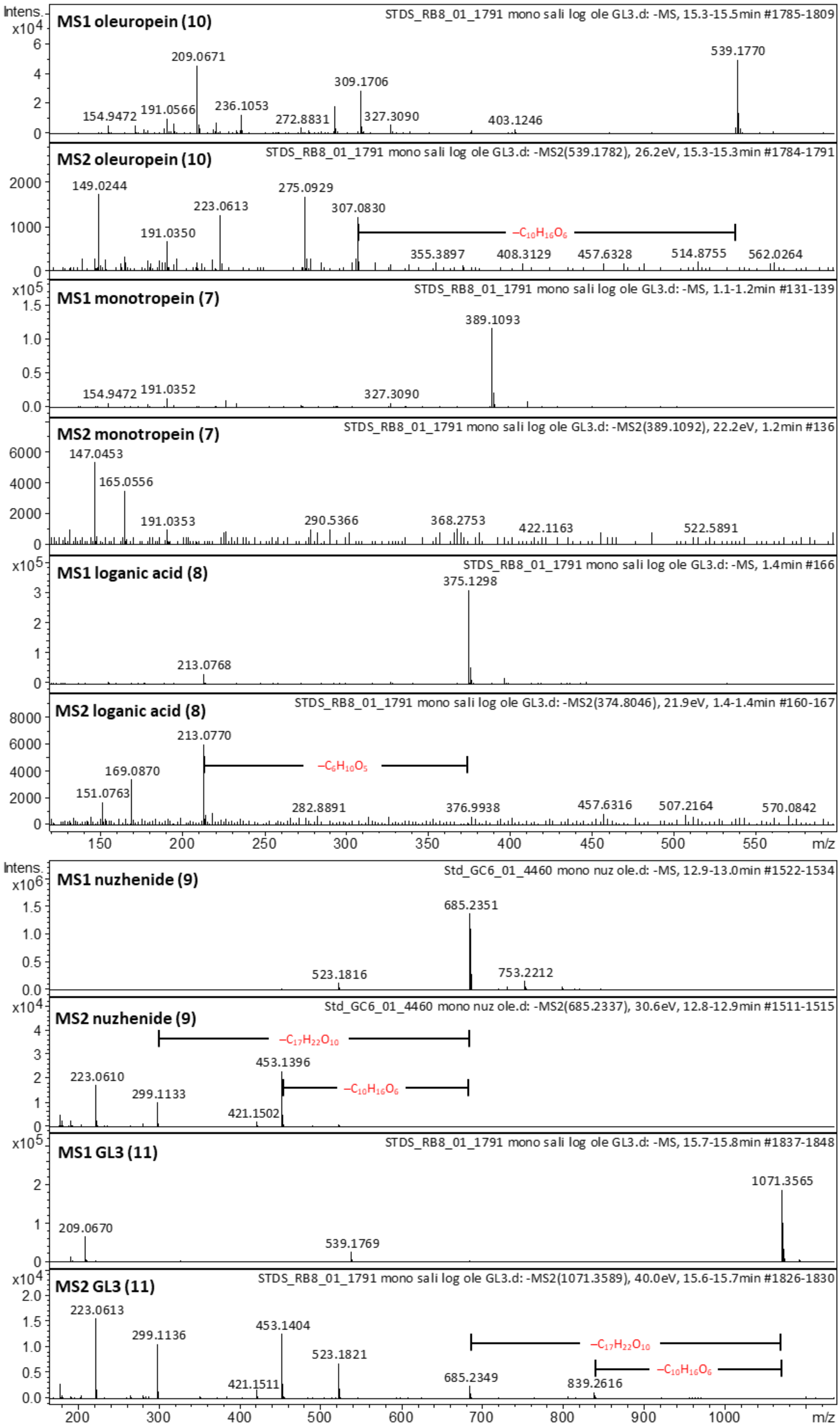

Supplementary Figure S8 – Negative ion mode MS1 and MS2 spectra of iridoid glycoside standards used in this study.

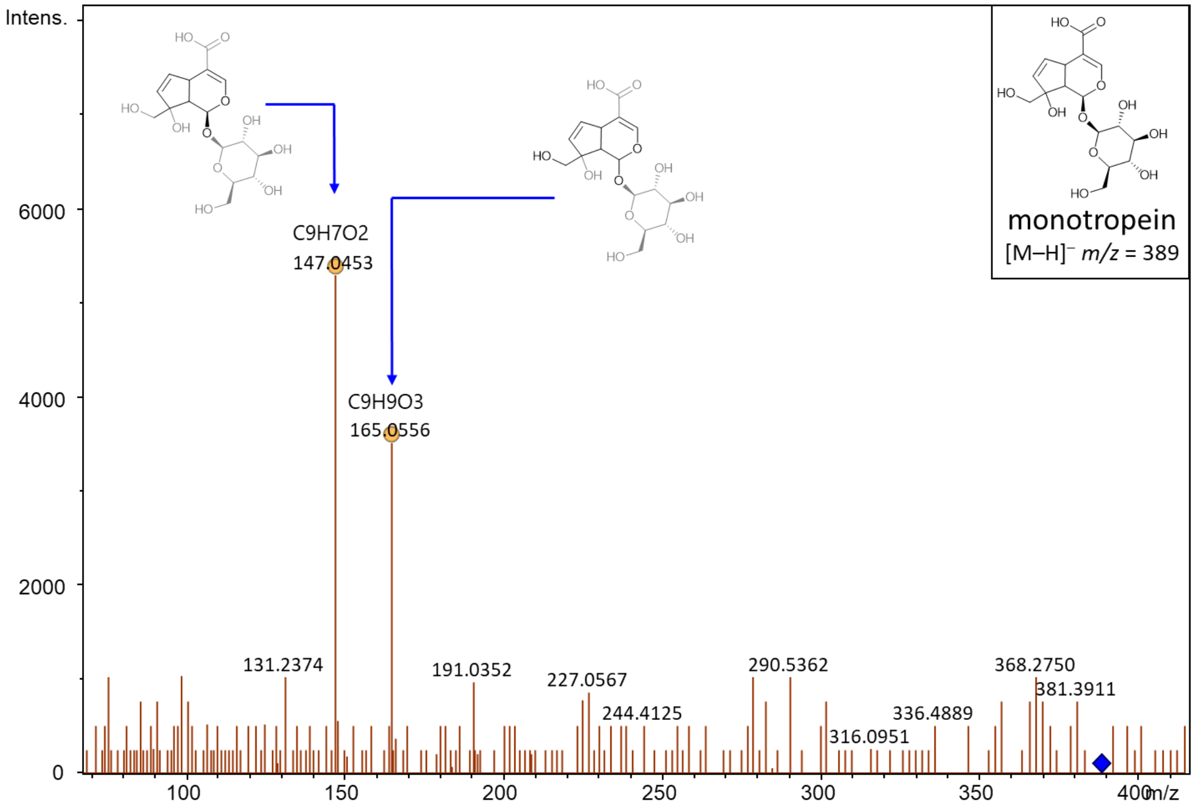

Supplementary Figure S9 – Negative ion mode MS2 spectrum of [M–H]$^{-}$ of (**7**) monotropein standard.

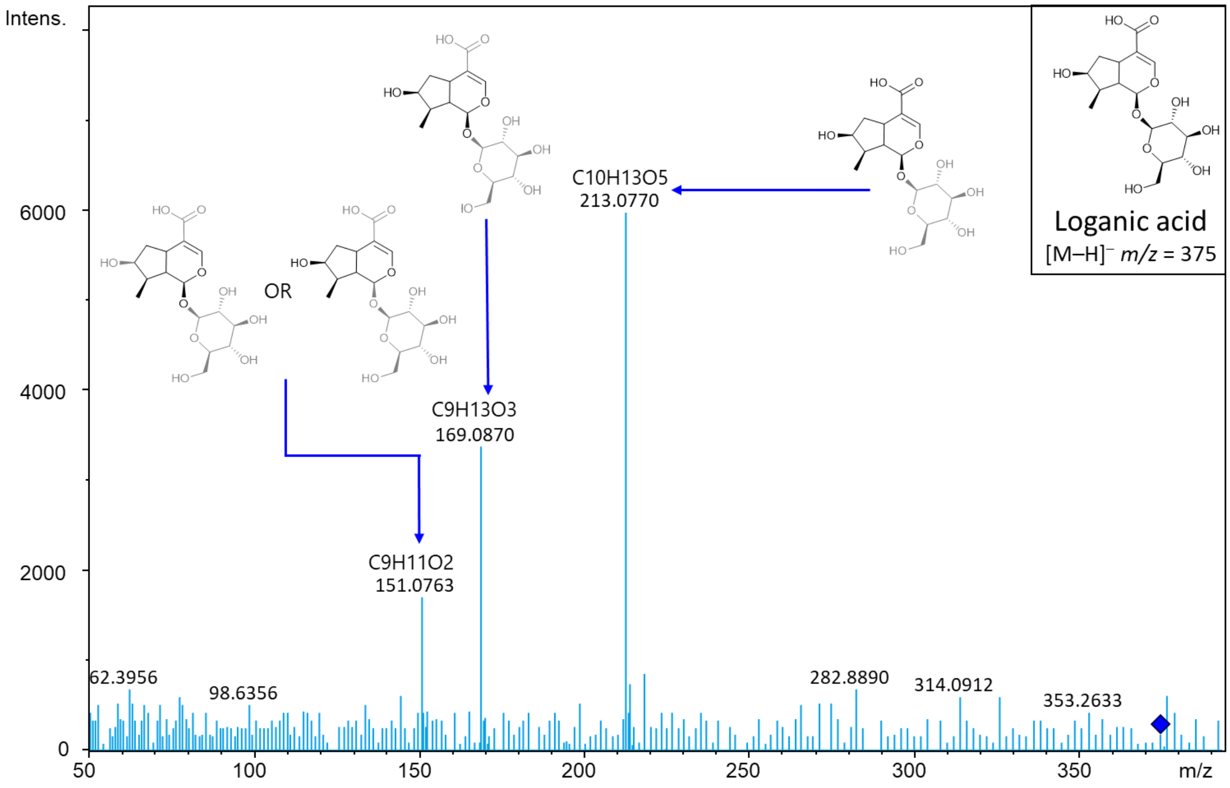

Supplementary Figure S10 – Negative ion mode MS2 spectrum of [M–H]$^{-}$ of (**8**) loganic acid standard.

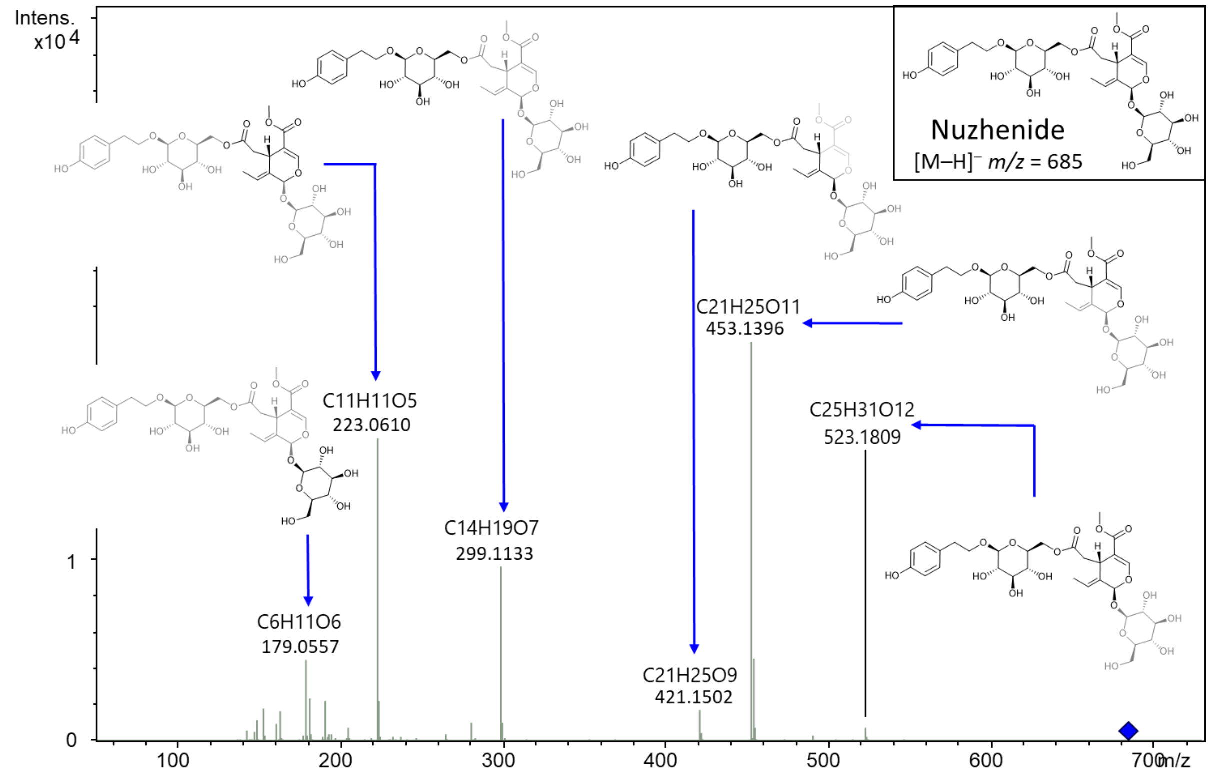

Supplementary Figure S11 – Negative ion mode MS2 spectrum of [M–H]$^{-}$ of (**9**) nuzhenide standard.

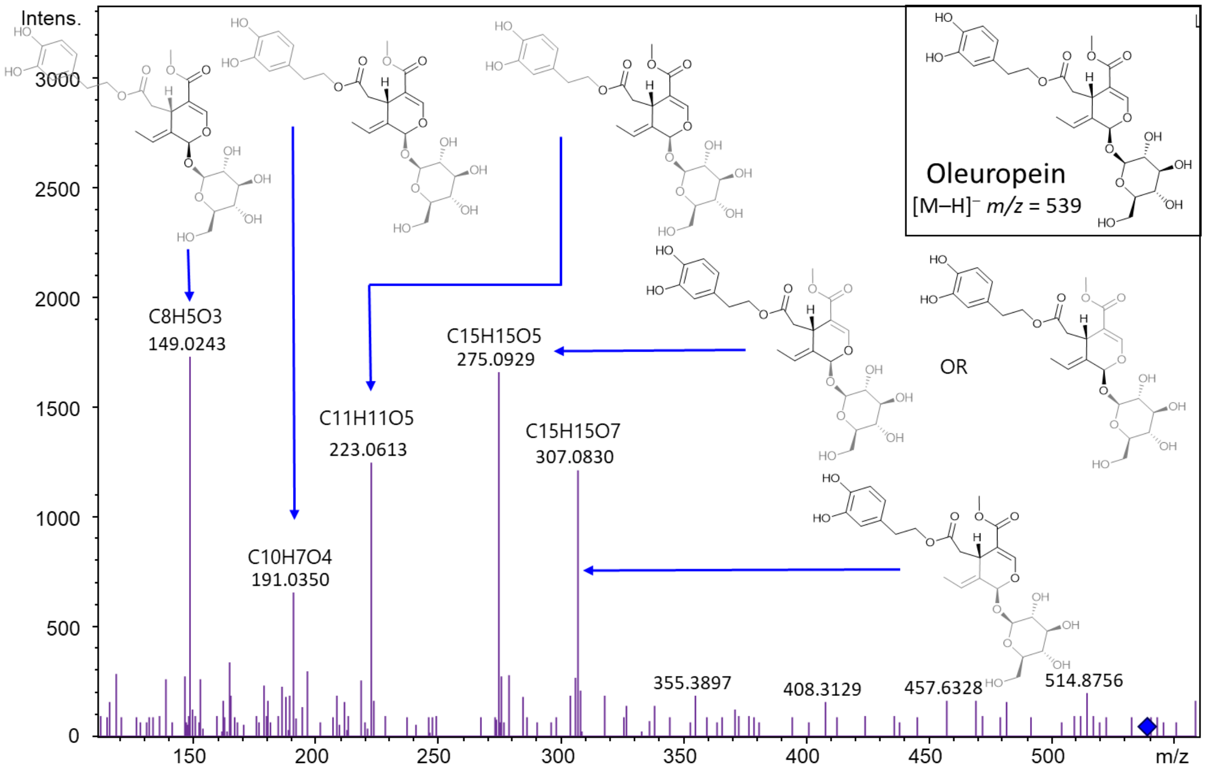

Supplementary Figure S12 – Negative ion mode MS2 spectrum of [M–H]$^{-}$ of (**10**) oleuropein standard.

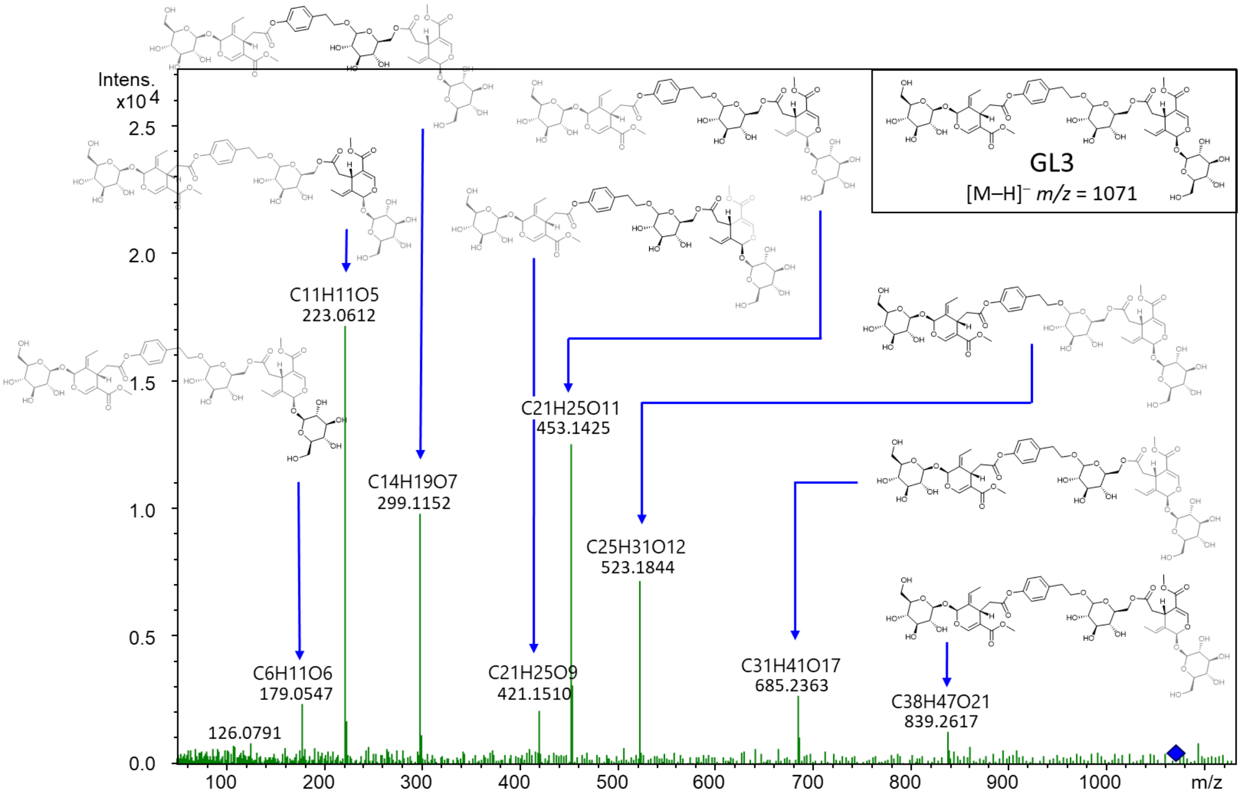

Supplementary Figure S13 – Negative ion mode MS2 spectrum of [M–H]$^{-}$ of (**11**) GL3 standard.

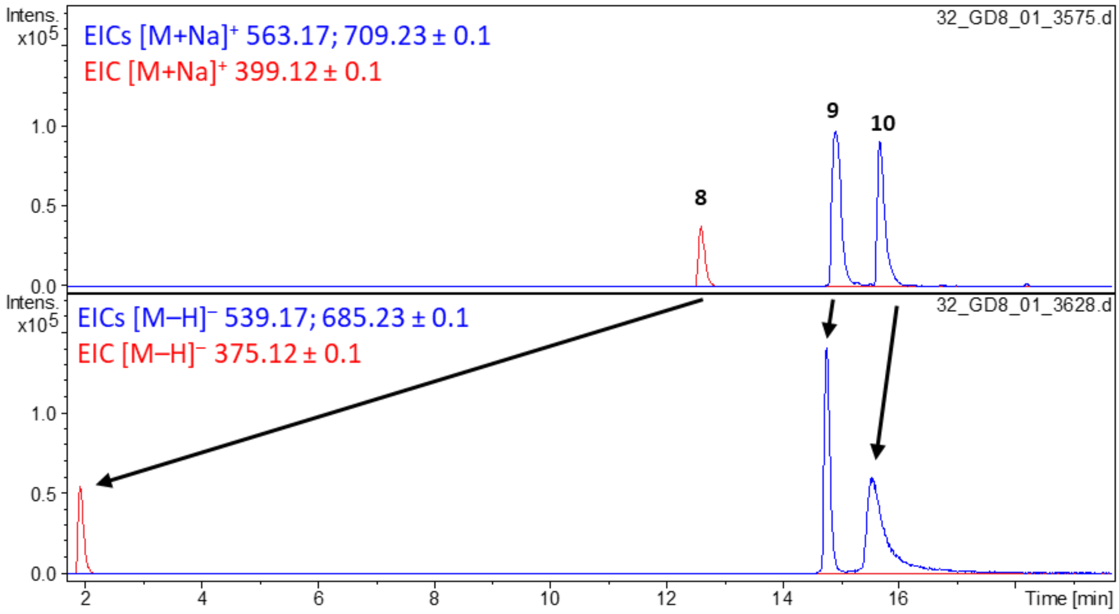

Supplementary Figure S14 – Extracted ion chromatograms for [M+Na]$^{+}$ and [M–H]$^{-}$ adducts of loganic acid **8**, nuzhenide **9** and oleuropein **10** standards in positive ion mode (upper panel) and negative ion mode (lower panel). Loganic acid **8** elutes much earlier with the eluent used for negative ion mode.

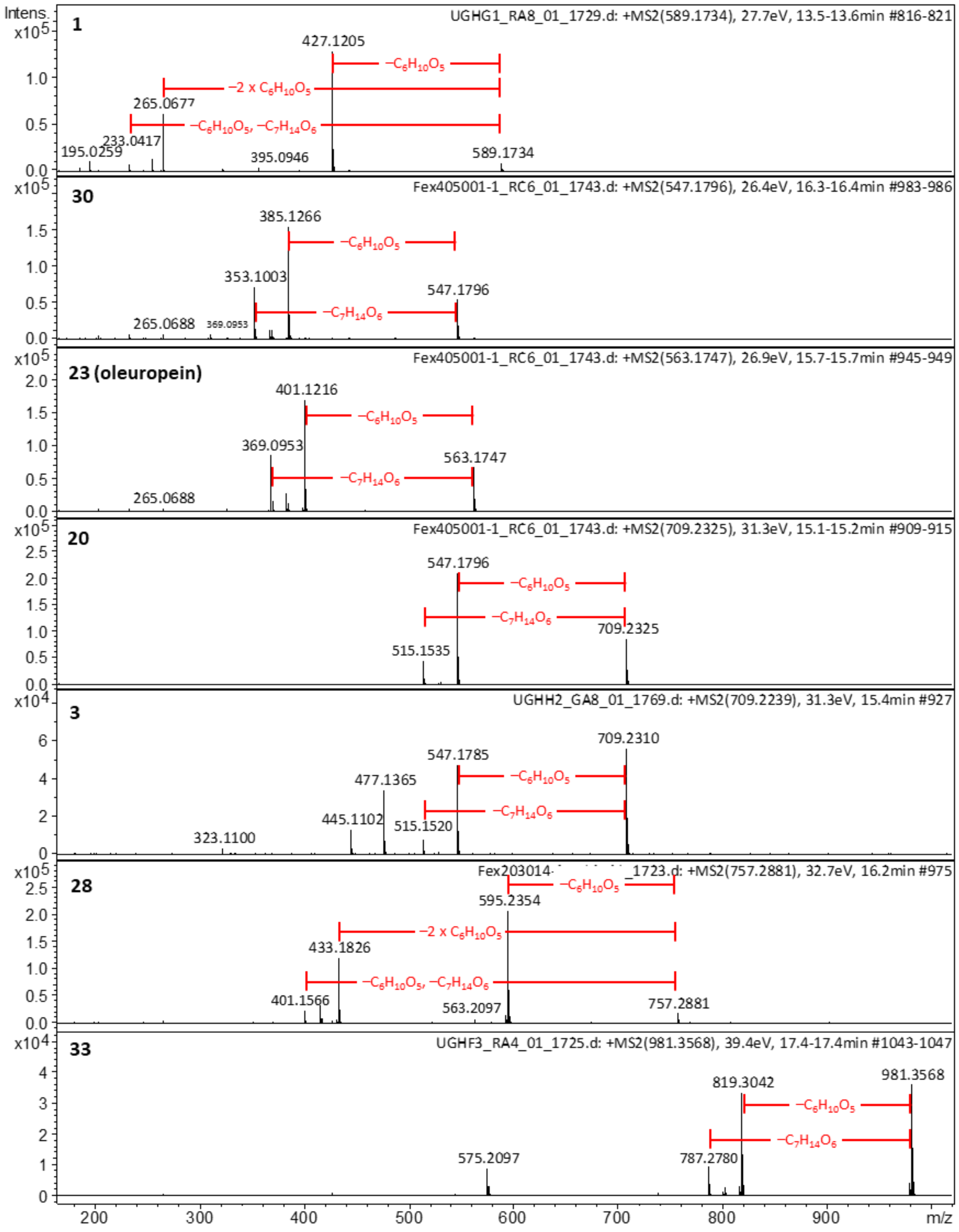

Supplementary Figure S15 – Example positive ion mode MS2 spectra for $[M+Na]^+$ adducts of secoiridoid glycosides identified in ash leaf extracts. Neutral losses of $C_6H_{10}O_5$ and $C_7H_{14}O_6$ are highlighted in red.

Supplementary Table S1 – Positive ion mode MS2 data for [M+Na]$^+$ adducts of iridoid glycosides observed in this study and neural losses of 162 and 194

Compound	*Rt/min*	*MS1 [M+Na]$^+$* m/z *observed*	m/z *calculated*	*Formula [M+Na]$^+$*	*MS2 [M–$C_6H_{10}O_5$+Na]$^+$* m/z *observed*	m/z *calculated*	*MS2 [M–$C_7H_{14}O_6$+Na]$^+$* m/z *observed*	m/z *calculated*	*assignment*	*refs*
iridoid glycoside standards										
monotropein(**7**)	1.2	413.1057	413.1054	$C_{16}H_{22}NaO_{11}$	251.0529	251.0526	–	–	monotropein	
loganic acid(**8**)	12.6	399.1256	399.1262	$C_{16}H_{24}NaO_{10}$	237.0734	237.0733	–	–	loganic acid	
nuzhenide(**9**)	14.9	709.2293	709.2314	$C_{31}H_{42}NaO_{17}$	547.1767	547.1786	515.1506	515.1524	nuzhenide	
oleuropein(**10**)	15.7	563.1715	563.1735	$C_{25}H_{32}NaO_{13}$	401.1201	401.1204	369.0940	369.0942	oleuropein	
GL3(**11**)	15.8	1095.3500	1095.3492	$C_{48}H_{64}NaO_{27}$	933.2980	933.2999	901.2723	901.2737	GL3	
putative iridoid glycosides in ash leaf extracts										
**12**	12.0	443.1157	443.1160	$C_{17}H_{24}NaO_{12}$	281.0628	281.0632	249.0366	249.0370	10-hydroxyoleoside 11-methyl ester	2
**13**	12.4	427.1213	427.1211	$C_{17}H_{24}NaO_{11}$	265.0675	265.0683	233.0412	233.0420	oleoside methyl ester	3, 4
**14**	–	not observed							demethyloleuropein	5, 6
**15**	13.1	589.1729	589.1739	$C_{23}H_{34}NaO_{16}$	427.1208	427.1211	395.0943	395.0949	methylglucooleoside or isomer	3
**16**	–	not observed							demethylligustroside	6
**1**/P2/N2	13.5	589.1728	589.1739	$C_{23}H_{34}NaO_{16}$	427.1208	427.1211	395.0945	395.0949	methylglucooleoside or isomer	1, 3
**17**	14.8	579.1680	579.1684	$C_{25}H_{32}NaO_{14}$	417.1152	417.1156	385.0890	385.0894	10-hydroxyoleuropein	7
**18**	14.9	709.2311	709.2314	$C_{31}H_{42}NaO_{17}$	547.1778	547.1786	515.1516	515.1524	nuzhenide isomer	5
**19**	14.9	441.1376	441.1367	$C_{18}H_{26}NaO_{11}$	279.0844	279.0839	247.0582	247.0577	oleoside dimethyl ester	3, 8
**20**	15.0	709.2323	709.2314	$C_{31}H_{42}NaO_{17}$	547.1796	547.1786	515.1535	515.1524	nuzhenide isomer	5
**2**/P5	15.0	709.2311	709.2314	$C_{31}H_{42}NaO_{17}$	547.1785	547.1786	515.1523	515.1524	nuzhenide isomer	1, 5
**21**	15.3	563.1729	563.1735	$C_{25}H_{32}NaO_{13}$	401.1206	401.1207	369.0943	369.0945	10-hydroxyligustroside	3
**3**/P1/N4	15.4	709.2308	709.2314	$C_{31}H_{42}NaO_{17}$	547.1784	547.1786	515.1517	515.1524	nuzhenide isomer	1
**22**	15.5	1033.3161	1033.3159	$C_{46}H_{58}NaO_{25}$	871.2526	871.2531	839.2363	839.2369	oleoacetoside or isomer	5, 9, 10
**23**	15.7	563.1725	563.1735	$C_{25}H_{32}NaO_{13}$	401.1204	401.1207	369.0942	369.0945	oleuropein (**10**)	3
**24**	15.9	547.1782	547.1786	$C_{25}H_{32}NaO_{12}$	385.1257	385.1258	353.0993	353.0996	excelsioside	3
**25**	16.0	741.2935	741.2940	$C_{33}H_{50}NaO_{17}$	579.2409	579.2412	547.2148	547.2150	jashemsloside C/D isomer	11
**26**	16.1	625.2092	625.2103	$C_{27}H_{38}NaO_{15}$	463.1570	463.1575	431.1307	431.1313	2”-epi-frameroside	12
**27**	16.2	783.3401	783.3410	$C_{36}H_{56}NaO_{17}$	621.2874	621.2881	589.2613	589.2619	isomer of (9) from *R. glutinosa*	13
**28**	16.2	757.2878	757.2889	$C_{33}H_{50}NaO_{18}$	595.2359	595.2361	563.2095	563.2099	jaspofoliamoside A or isomer	9
**29**	16.3	933.2989	933.2999	$C_{42}H_{54}NaO_{22}$	771.2457	771.2471	739.2193	739.2209	GL5 or isomer	3, 5, 14
**30**	16.3	547.1783	547.1786	$C_{25}H_{32}NaO_{12}$	385.1259	385.1258	353.0996	353.0996	ligustroside	3
**31**	16.7	933.3004	933.2999	$C_{42}H_{54}NaO_{22}$	771.2481	771.2471	739.2216	739.2209	GL5 or isomer	3, 5, 14
**4**/P7	17.1	933.2987	933.2999	$C_{42}H_{54}NaO_{22}$	771.2463	771.2471	739.2208	739.2209	GL5 or isomer	1, 3, 5, 14
**32**	17.1	967.3426	967.3418	$C_{43}H_{60}NaO_{23}$	805.2874	805.2889	773.2612	773.2627	pulosarioside isomer	15
**33**	17.4	981.3565	981.3574	$C_{44}H_{62}NaO_{23}$	819.3042	819.3046	787.2780	787.2784	jaspofoliamoside E or isomer	16

Supplementary Table S2 – Positive ion mode data for [M–X+H]$^+$ peaks of iridoid glycosides arising from in-source fragmentation

Compound	*Rt/min*	*Formula*	*predominant MS1 [M–X+H]$^+$ peak*			*neutral loss*	*proposed assignment*
		[M+Na]$^+$	m/z *observed*	m/z *calculated*	*formula*		
iridoid glycoside standards							
monotropein(**7**)	1.2	$C_{16}H_{22}NaO_{11}$	211.0602	211.0601	$C_{10}H_{11}O_5$	180	hexose (glucose)
loganic acid(**8**)	12.6	$C_{16}H_{24}NaO_{10}$	215.0910	215.0914	$C_{10}H_{15}O_5$	162	glucosyl
nuzhenide(**9**)	14.9	$C_{31}H_{42}NaO_{17}$	507.1844	507.1837	$C_{25}H_{31}O_{11}$	180	hexose (glucose)
oleuropein(**10**)	15.7	$C_{25}H_{32}NaO_{13}$	361.1267	361.1282	$C_{19}H_{21}O_7$	180	hexose (glucose)
GL3(**11**)	15.8	$C_{48}H_{64}NaO_{27}$	713.2427	713.2440	$C_{36}H_{41}O_{15}$	360	2 x hexose (glucose)
putative iridoid glycosides in ash leaf extracts							
**12**	12.0	$C_{17}H_{24}NaO_{12}$	241.0710	241.0707	$C_{11}H_{13}O_6$	180	hexose
**13**	12.4	$C_{17}H_{24}NaO_{11}$	243.0854	243.0863	$C_{11}H_{15}O_6$	162	glucosyl
**14**	not observed in positive ion mode						
**15**	13.1	$C_{23}H_{34}NaO_{16}$	225.0754	225.0757	$C_{11}H_{13}O_5$	342	hexose+glucosyl
**16**	not observed in positive ion mode						
**1**/P2/N2	13.5	$C_{23}H_{34}NaO_{16}$	225.0752	225.0757	$C_{11}H_{13}O_5$	342	hexose+glucosyl
**17**	14.8	$C_{25}H_{32}NaO_{14}$	377.1230	377.1231	$C_{19}H_{21}O_8$	180	hexose
**18**	14.9	$C_{31}H_{42}NaO_{17}$	345.1330	345.1333	$C_{19}H_{21}O_6$	342	hexose+glucosyl
**19**	14.9	$C_{18}H_{26}NaO_{11}$	239.0919	239.0914	$C_{12}H_{15}O_5$	180	hexose
**20**	15.0	$C_{31}H_{42}NaO_{17}$	345.1338	345.1333	$C_{19}H_{21}O_6$	342	hexose+glucosyl
**2**/P5	15.0	$C_{31}H_{42}NaO_{17}$	345.1331	345.1333	$C_{19}H_{21}O_6$	342	hexose+glucosyl
**21**	15.3	$C_{25}H_{32}NaO_{13}$	361.1278	361.1282	$C_{19}H_{21}O_7$	180	hexose
**3**/P1/N4	15.4	$C_{31}H_{42}NaO_{17}$	363.1434	363.1438	$C_{19}H_{23}O_7$	324	glucosyl+glucosyl
**22**	15.5	$C_{46}H_{58}NaO_{25}$	831.2692	831.2706	$C_{40}H_{47}O_{19}$	180	hexose
**23**	15.7	$C_{25}H_{32}NaO_{13}$	361.1275	361.1282	$C_{19}H_{21}O_7$	180	hexose
**24**	15.9	$C_{25}H_{32}NaO_{12}$	345.1331	345.1333	$C_{19}H_{21}O_6$	180	hexose
**25**	16.0	$C_{33}H_{50}NaO_{17}$	not obs.	not obs.		n/a	n/a
**26**	16.1	$C_{27}H_{38}NaO_{15}$	423.1642	423.1650	$C_{21}H_{27}O_9$	180	hexose
**27**	16.2	$C_{36}H_{56}NaO_{17}$	581.2951	581.2956	$C_{30}H_{45}O_{11}$	180	hexose
**28**	16.2	$C_{33}H_{50}NaO_{18}$	393.1902	393.1884	$C_{21}H_{29}O_7$	342	hexose+glucosyl
**29**	16.3	$C_{42}H_{54}NaO_{22}$	731.2537	731.2546	$C_{36}H_{43}O_{16}$	180	hexose
**30**	16.3	$C_{25}H_{32}NaO_{12}$	345.1332	345.1333	$C_{19}H_{21}O_6$	180	hexose
**31**	16.7	$C_{42}H_{54}NaO_{22}$	731.2552	731.2546	$C_{36}H_{43}O_{16}$	180	hexose
			551.1918	551.1912	$C_{30}H_{31}O_{10}$	360	2 x hexose
**4**/P7	17.1	$C_{42}H_{54}NaO_{22}$	731.2534	731.2546	$C_{36}H_{43}O_{16}$	180	hexose
**32**	17.1	$C_{43}H_{60}NaO_{23}$	765.2974	765.2964	$C_{37}H_{49}O_{17}$	180	hexose
**33**	17.4	$C_{44}H_{62}NaO_{23}$	779.3116	779.3121	$C_{38}H_{51}O_{17}$	180	hexose

Supplementary Table S3 – Negative ion mode MS2 data for iridoid glycosides identified and fragments corresponding to losses of 232, 264 and 386 Da

Compound	*Rt/min*	*[M–H]⁻* m/z *observed*	*[M–H]⁻* m/z *calculated*	*[M–H]⁻ formula*	*MS2 [M–$C_{10}H_{16}O_6$–H]⁻* m/z *observed*	m/z *calculated*	*MS2 [M–$C_{10}H_{16}O_8$–H]⁻* m/z *observed*	m/z *calculated*	*MS2 [M–$C_{17}H_{22}O_{10}$–H]⁻* m/z *observed*	m/z *calculated*
iridoid glycoside standards										
monotropein(**7**)	1.2	389.1093	389.1089	$C_{16}H_{21}O_{11}$	not obs.	–	–	–	–	–
loganic acid(**8**)*	1.4	375.1297	375.1297	$C_{16}H_{23}O_{10}$	–	–	–	–	–	–
nuzhenide(**9**)	14.9	685.2348	685.2349	$C_{31}H_{41}O_{17}$	453.1396	453.1402	421.1502	421.1504	299.1133	299.1136
oleuropein(**10**)	15.4	539.1771	539.1770	$C_{25}H_{31}O_{13}$	307.0830	307.0823	275.0929	275.0925	-	-
GL3(**11**)	15.6	1071.3559	1071.3562	$C_{48}H_{63}O_{27}$	839.2617	839.2615	–	–	685.2350	685.2349
**6**/N3	13.3	449.1310	449.1301	$C_{18}H_{25}O_{13}$	–	–	n/a	n/a	–	–
putative iridoid glycosides in ash leaf extracts										
**12**	-	*m/z* 419	not detected	not detected	n/a	n/a	n/a	n/a	n/a	n/a
**13***	4.7	403.1241	403.1246	$C_{17}H_{23}O_{11}$	–	–	–	–	–	–
**14**	12.7	525.1606	525.1614	$C_{24}H_{29}O_{13}$	–	–	–	–	–	–
**15**	13.1	565.1782	565.1774	$C_{23}H_{33}O_{16}$	–	–	–	–	179.0563	179.0561
**16**	13.3	509.1658	509.1664	$C_{24}H_{29}O_{12}$	277.0719	277.0718	–	–	–	–
**1**/P2/N2	13.5	565.1778	565.1774	$C_{23}H_{33}O_{16}$	–	–	–	–	179.0564	179.0561
**17**	14.3	555.1715	555.1719	$C_{25}H_{31}O_{14}$	–	–	307.0813	307.0823	–	–
**18**	14.9	685.2352	685.2349	$C_{31}H_{41}O_{17}$	453.1400	453.1402	–	421.1504	–	–
**19**	14.9	417.1406	417.1402	$C_{18}H_{25}O_{11}$	185.0457	185.0455	153.0554	153.0557	–	–
**2**/P5	15.1	685.2352	685.2349	$C_{31}H_{41}O_{17}$	453.1411	453.1402	421.1503	421.1504	299.1149	299.1136
**20**	15.1	685.2353	685.2349	$C_{31}H_{41}O_{17}$	453.1398	453.1402	421.1524	421.1504	299.1137	299.1136
**21**	15.1	539.1769	539.1170	$C_{25}H_{31}O_{13}$	–	–	275.0924	275.0925	-	-
**3**/P1/N4	15.3	685.2346	685.2349	$C_{31}H_{41}O_{17}$	453.1404	453.1402	421.1498	421.1504	299.1139	299.1136
**22***	14.2	1009.3186	1009.3194	$C_{46}H_{57}O_{25}$	777.2262	777.2248	745.2355	745.2349	623.1980	623.1981
**23**	15.3	539.1780	539.1170	$C_{25}H_{31}O_{13}$	307.0825	307.0823	275.0562	275.0561	153.0557	153.0557
**24**	15.9	523.1813	523.1821	$C_{25}H_{31}O_{12}$	–	–	–	–	137.0602	137.0608
**25**	15.9	717.2967	717.2975	$C_{33}H_{49}O_{17}$	485.2022	485.2028	453.2127	453.2130	331.1755	331.1762
**26***	12.0	601.2137	601.2138	$C_{27}H_{37}O_{15}$	–	–	–	–	–	–
**27**	16.1	759.3486	759.3445	$C_{36}H_{55}O_{17}$	527.2502	527.2498	495.2605	495.2600	–	–
**28**	16.1	733.2919	733.2924	$C_{33}H_{49}O_{18}$	–	–	–	–	347.1713	347.1711
**29**	16.1	909.3031	909.3034	$C_{42}H_{53}O_{22}$	–	–	–	–	–	–
**30**	16.1	523.1824	523.1821	$C_{25}H_{31}O_{12}$	291.0878	291.0874	259.0978	259.0976	–	–
**31**	16.6	909.3017	909.3034	$C_{42}H_{53}O_{22}$	677.2092	677.2087	–	–	523.1840	523.1821
**4**/P7	17.0	909.3019	909.3034	$C_{42}H_{53}O_{22}$	677.2096	677.2087	–	–	–	–
**32***	14.3	943.3451	943.3453	$C_{43}H_{59}O_{23}$	–	–	–	–	557.2241	557.2240
**33**	17.2	957.3613	957.3609	$C_{44}H_{61}O_{23}$	725.2675	725.2662	693.2780	693.2764	571.2402	571.2396

*indicates compounds with significant retention time shifts (>2 min earlier) in negative mode compared to positive ion mode due to different mobile phase

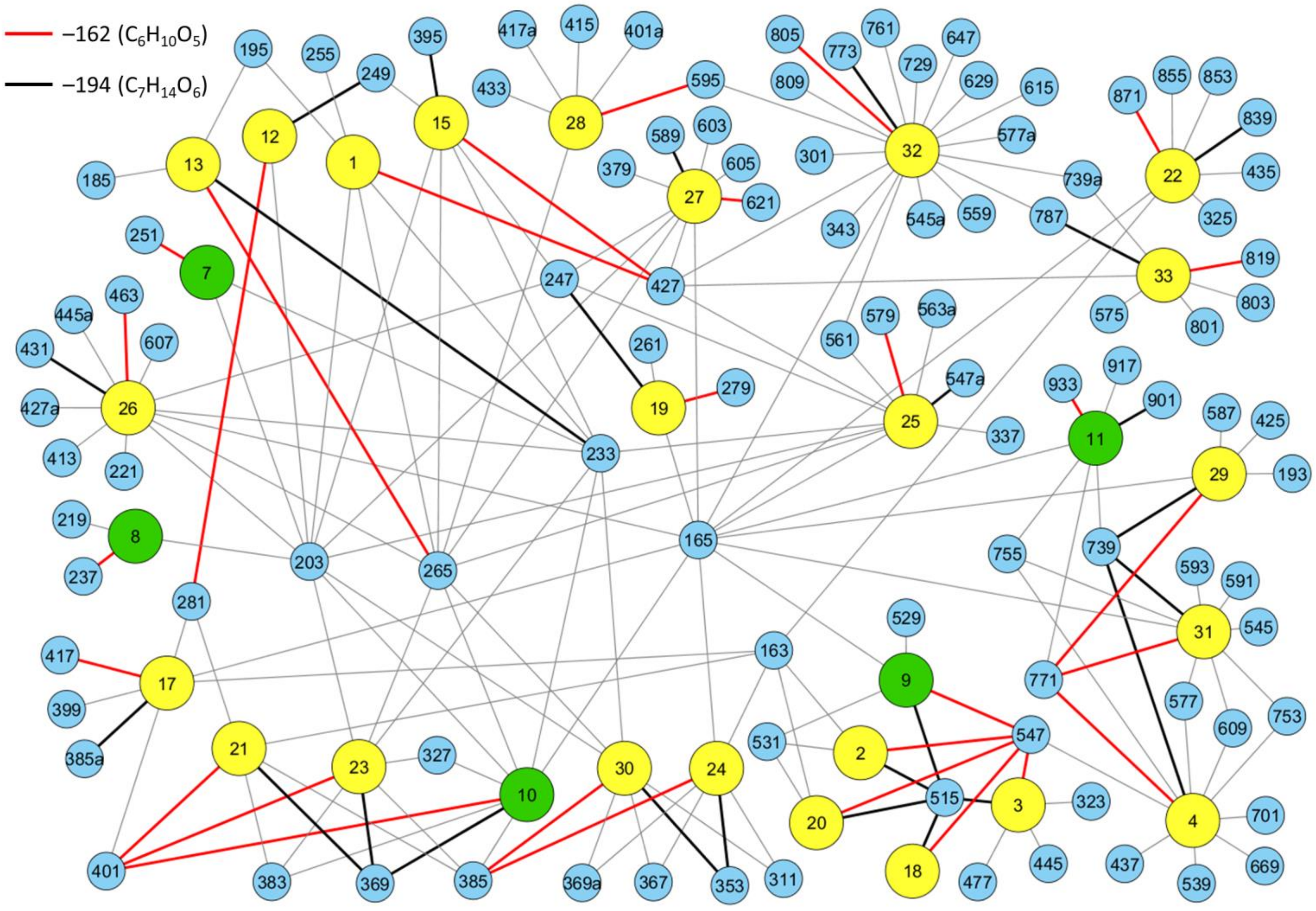

***Supplementary Figure S16* –** Molecular network showing fragment ions in [M+Na]$^+$ MS2 spectra of **1-33** observed in ash leaf extracts (yellow nodes). Iridoid glycoside standards monotropein (**7**), loganic acid (**8**), nuzhenide (**9**), oleuropein (**10**) and GL3 (**11**) are denoted by green nodes. Fragment ions (blue nodes) with intensities >3% of base peak are included. Fragment ions denoted Xa, Xb, have different molecular formulae as determined by HRMS/MS.

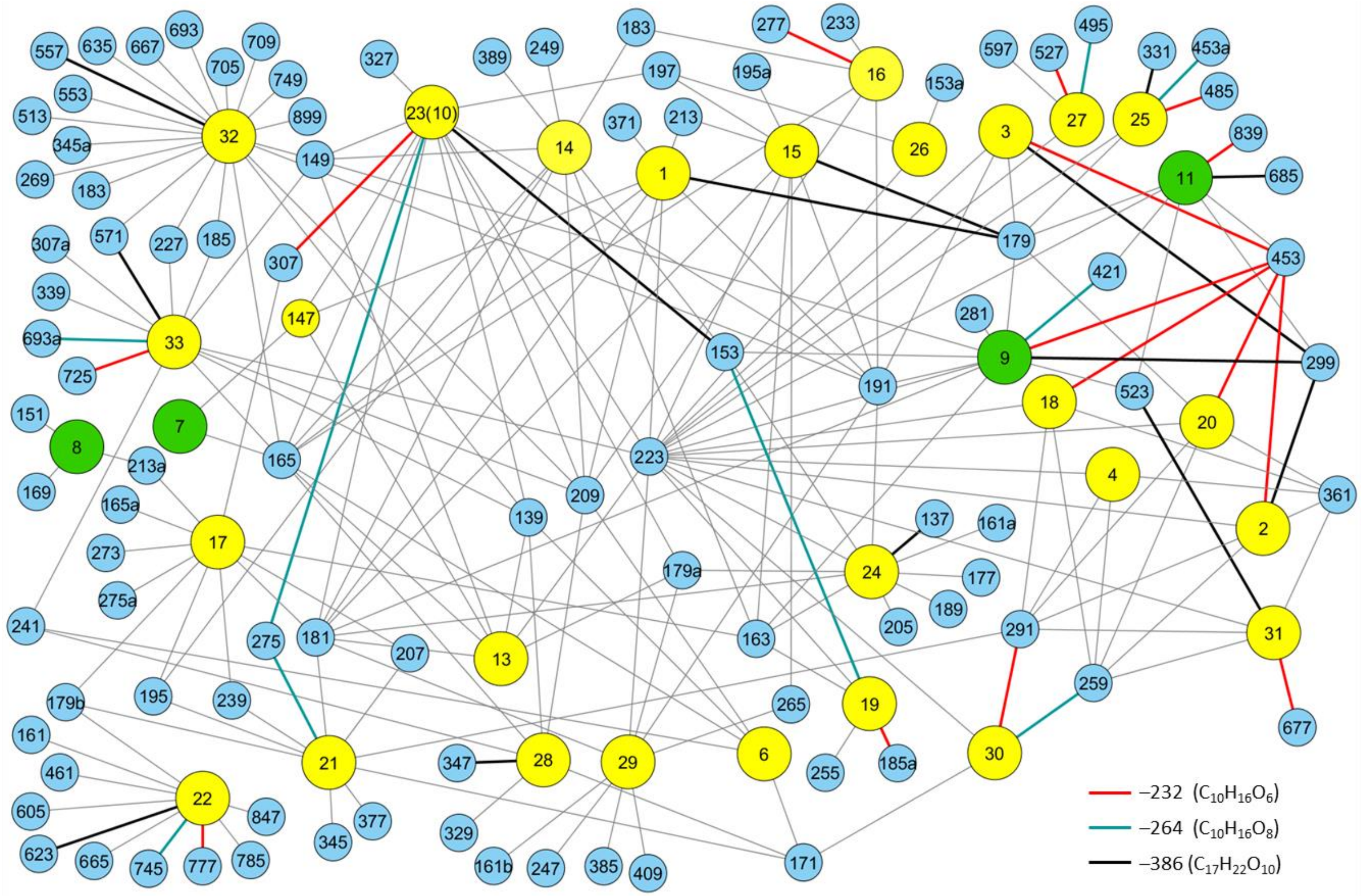

Supplementary Figure S17 – Molecular network of fragment ions from MS2 spectra of $[M–H]^-$ of **1-33.** Compounds observed in ash leaf extracts are denoted by yellow nodes. Iridoid glycoside standards monotropein (**7**), loganic acid (**8**), nuzhenide (**9**) and GL3 (**11**) are denoted by green nodes. Fragment ion (blue) *m/z* given to nearest integer for clarity. Only peak intensities >10% intensity of base peak are included. Fragment ions that arise from neutral losses of 232 ($C_{10}H_{16}O_6$), 264 ($C_{10}H_{16}O_8$) and 386 ($C_{17}H_{22}O_{10}$) are highlighted. Fragment ions denoted Xa, Xb, have different molecular formulae as determined by HRMS/MS.

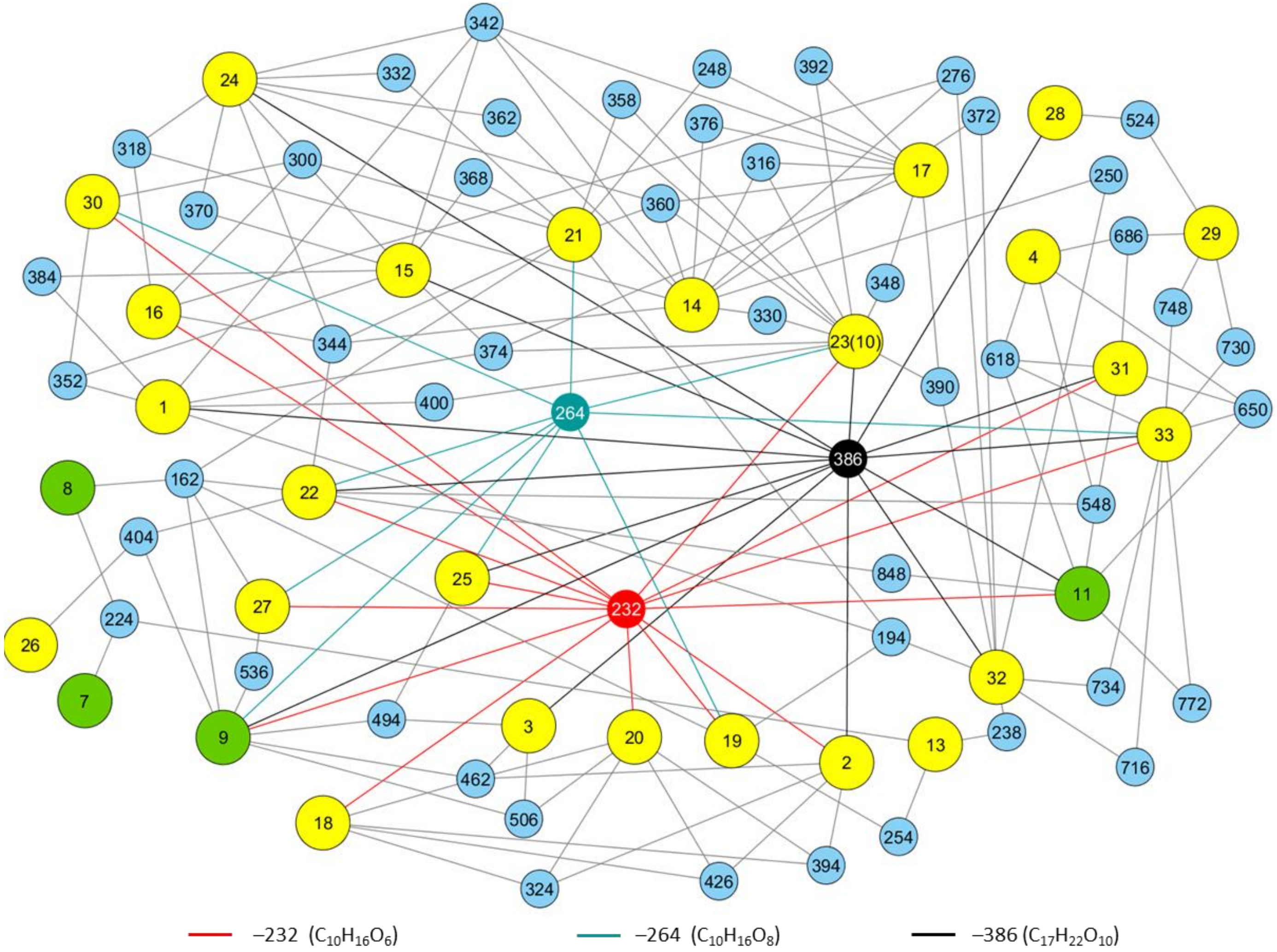

Supplementary Figure S18 – Molecular network of neutral losses from MS2 spectra of $[M–H]^-$ of **1-33.** Compounds observed in ash leaf extracts are denoted by yellow nodes. Iridoid glycoside standards monotropein (**7**), loganic acid (**8**), nuzhenide (**9**) and GL3 (**11**) are denoted by green nodes. Shared neutral losses (blue) *m/z* given to nearest integer for clarity. Only peak intensities >10% intensity of base peak are included. Neutral losses of 232 ($C_{10}H_{16}O_6$), 264 ($C_{10}H_{16}O_8$) and 386 ($C_{17}H_{22}O_{10}$) are highlighted.

***Supplementary Table S4* – Shared fragment ions in MS2 spectra of 1-33 in positive ion mode**

fragment m/z *observed*	*calculated*	*formula*	*putative assignment*	*no. of compounds*	*notes*	*refs*
163.0392	163.0390	$C_9H_7O_3$	[secoiridoid aglycone core<br>$-CH_2O_2-CH_3OH-H_2+H]^+$	6	**2**, **20**, **17**, **21**, **22**, **24**	
165.0550	165.0546	$C_9H_9O_3$	[secoiridoid aglycone core<br>$-CH_3OH-CH_2O_2+H]^+$	13		17
195.0259	195.0264	$C_7H_8O_5Na$	[secoiridoid aglycone core$-C_4H_6O+Na]^+$	2	**1** and **13** only	
203.0529	203.0526	$C_6H_{12}O_6Na$	$[hexose+Na]^+$	11		
233.0426	233.0420	$C_{10}H_{10}O_5Na$	[secoiridoid aglycone core$-CH_3OH+Na]^+$<br>[monotropein aglycone$-H_2O+Na]^+$	8	 monotropein(**7**)	
247.0580	247.0577	$C_{11}H_{12}O_5Na$	[secoiridoid aglycone core$+H_2O+Na]^+$	5	**19**, **15**, **25**, **26**, **27**	
249.0730	249.0733	$C_{11}H_{14}O_5Na$	[secoiridoid aglycone core$-O+Na]^+$	2	**12** and **15**	
265.0685	265.0683	$C_{11}H_{14}O_6Na$	[secoiridoid aglycone core$+Na]^+$	10		
281.0636	281.0632	$C_{11}H_{14}O_7Na$	[secoiridoid core$+O+Na]^+$	3	**12**, **17**, **21**	
311.0900	311.0890	$C_{16}H_{16}O_5Na$	$[M-C_6H_{10}O_5-C_3H_6O_2+Na]^+$	2	**24** and **30**	
353.1005	353.0996	$C_{18}H_{18}O_6Na$	$[M-C_7H_{14}O_6+Na]^+$	2	**24** and **30**	
367.1158	367.1152	$C_{19}H_{20}O_6Na$	$[M-C_6H_{12}O_6+Na]^+$	2	**24** and **30**	
369.0951	369.0945	$C_{18}H_{18}O_7Na$	$[M-C_7H_{14}O_6+Na]^+$	2	**21** and<br>**23**(oleuropein;**10**)	
369.1316 (a)	369.1309	$C_{19}H_{22}O_6Na$	$[M-C_6H_{10}O_6+Na]^+$	2	**24** and **30**	
383.1110	383.1101	$C_{19}H_{20}O_7Na$	$[M-C_6H_{12}O_6+Na]^+$	2	**21** and<br>**23**(oleuropein;**10**)	
385.1265	385.1258	$C_{19}H_{22}O_7Na$	$[M-C_6H_{10}O_6+Na]^+$<br>$[M-C_6H_{10}O_5+Na]^+$	4	**21**,<br>**23**(oleuropein;**10**)<br>**24** and **30**	
401.1215	401.1207	$C_{19}H_{22}O_8Na$	$[M-C_6H_{10}O_5+Na]^+$<br>$[M-C_6H_{10}O_6+Na]^+$	3	**23**(oleuropein;**10**)<br>**17**, **21**	
427.1209	427.1211	$C_{17}H_{24}O_{11}Na$	$[M-C_6H_{10}O_5+Na]^+$<br>[oleoside methyl ester$+Na]^+$	6	**1** and **15**<br>**25**, **27**, **32**, **33**	
515.1517	515.1533	$C_{24}H_{28}O_{11}Na$	$[M-C_7H_{14}O_6+Na]^+$	4	**2**, **3**, **18**, **20** and<br>nuzhenide(**9**)	
531.1848	531.1837	$C_{25}H_{32}O_{11}Na$	$[M-C_6H_{10}O_6+Na]^+$	2	**2**, **20** and<br>nuzhenide(**9**)	
547.1784	547.1786	$C_{25}H_{32}O_{12}Na$	$[M-C_7H_{14}O_6+Na]^+$<br><br>$[M-C_{17}H_{22}O_{10}+Na]^+$	5	**2**, **3**, **18**, **20** and<br>nuzhenide(**9**)<br>**4**	
577.1683	577.1680	$C_{29}H_{30}O_{11}Na$	$[M-C_7H_{14}O_6-C_6H_{10}O_5+Na]^+$	2	**31** and **4**	
595.2344	595.2361	$C_{27}H_{40}O_{13}Na$	$[M-C_6H_{10}O_5+Na]^+$<br>$[M-C_{16}H_{20}O_{10}+Na]^+$	2	**28**<br>**32**	
609.1946	609.1942	$C_{30}H_{34}O_{12}Na$	$[M-2C_6H_{10}O_5+Na]^+$	2	**31** and **4**	
739.2216	739.2209	$C_{35}H_{40}O_{16}Na$	$[M-C_7H_{14}O_6+Na]^+$<br>$[M-C_6H_{10}O_5-C_7H_{14}O_6+Na]^+$	3	**31** and **4**<br>GL3 standard(**11**)	
739.2768 (a)	739.2784	$C_{33}H_{48}O_{17}Na$	$[M-C_{10}H_{12}O_6+Na]^+$<br>$[M-C_{11}H_{14}O_6+Na]^+$	2	**32**<br>**33**	
753.2369	753.2365	$C_{36}H_{42}O_{16}Na$	$[M-C_6H_{12}O_6+Na]^+$	2	**31** and **4**	
755.2510	755.2522	$C_{36}H_{44}O_{16}Na$	$[M-C_6H_{10}O_6+Na]^+$<br>$[M-C_6H_{10}O_5-C_6H_{10}O_6+Na]^+$	2	**31** and **4**<br>GL3 standard(**11**)	
771.2472	771.2471	$C_{36}H_{44}O_{17}Na$	$[M-C_6H_{10}O_5+Na]^+$<br>$[M-2C_6H_{10}O_5+Na]^+$	3	**31** and **4**<br>GL3 standard(**11**)	
787.2768	787.2784	$C_{37}H_{48}O_{17}Na$	$[M-C_6H_{12}O_6+Na]^+$<br>$[M-C_7H_{14}O_6+Na]^+$	2	**32**<br>**33**	

***Supplementary Table S5* – Summary of shared fragments in MS2 spectra of 1-33 in negative ion mode**

fragment ion m/z		*formula*	*putative assignment*	*no. of compounds*	*details*	*refs*
observed	*calculated*					
139.0030	139.0037	$C_6H_3O_4$	[secoiridoid aglycone core<br>$–C_4H_6O–CH_3OH–H]^–$	6	**13**, **23**(**10**), **32**, **28**, **33**, **6**	7
147.0451	147.0452	$C_9H_7O_2$	[secoiridoid aglycone core<br>$– CH_3OH–CO–2H_2O\text{-}H]^–$<br>$[M–C_6H_{10}O_5–CO_2–2H_2O–H]^–$	3	**23**(oleuropein;**10**), **1**, **13**<br><br>monotropein(**7**)	
149.0235	149.0244	$C_8H_5O_3$	[secoiridoid aglycone core<br>$–CH_3COOH–CH_3OH–H]^–$	5	**13**, **23**(**10**), **32**, **14**<br>nuzhenide(**9**)	7
153.0554	153.0557	$C_8H_9O_3$	[*m*-hydroxytyrosol–H]$^–$	4	**23**(**10**), **14**, **24**, **19**<br>nuzhenide(**9**)	5, 7
163.0396	163.0401	$C_9H_7O_3$	[secoiridoid aglycone core<br>$–CH_3COOH–H_2O\text{-}H]^–$	5	**14**, **15**, **17**, **19**, **24**,<br>nuzhenide(**9**)	
165.0554	165.0557	$C_9H_9O_3$	[secoiridoid aglycone core<br>$– CO_2–CH_3OH–H]^–$<br>$[M–C_6H_{10}O_5–CO_2–H_2O–H]^–$	9	**23**(oleuropein;**10**) and 8 other compounds<br>monotropein(**7**)	7
171.0279	171.0299	$C_7H_7O_5$	[secoiridoid aglycone core<br>$–C_4H_6O–H]^–$	4	**6**, **21**, **28**, **30**	
179.0563	179.0561	$C_6H_{11}O_6$	[hexose–H]$^–$	5	**1**, **15**, **3**, **20**, **25**<br>nuzhenide(**9**) and GL3(**11**)	18
179.0712(a)	179.0714	$C_{10}H_{11}O_3$	[secoiridoid aglycone core<br>$–H_2O–CO_2–H]^–$	4	**13**, **23**, **24**, **29**	18
179.0348(b)	179.0350	$C_9H_7O_4$	[secoiridoid core+O<br>$–H_2O–CH_3COOH–H]^–$<br>[caffeoyl–H]$^–$	3	**17**, **21**<br><br>**22**	 <br>5
181.0506	181.0506	$C_9H_9O_4$	[secoiridoid aglycone core<br>$–CH_3COOH–H]^–$	9	**1**, **13**, **14**, **15**, **17**, **21**, **23**, **24**, **29** nuzhenide(**9**)	7
185.1188	185.1183	$C_{10}H_{17}O_3$	[monoterpene fragment–H]$^–$	2	**32** and **33** only	
195.0663	195.0663	$C_{10}H_{11}O_4$	[secoiridoid aglycone core<br>$–CO–H_2O–H]^–$<br>$[M–C_6H_{10}O_6–C_8H_8O_3–H]^–$	3	**17**, **21**<br><br>**14**	
197.0820	197.0819	$C_{10}H_{13}O_4$	[secoiridoid aglycone core$– CO_2–H]^–$	3	**15**, **23**(**10**), **26**	5
207.0298	207.0299	$C_{10}H_7O_5$	[secoiridoid aglycone core+O<br>$–CH_3OH–H_2O–H]^–$	2	**17** and **21**	
209.0457	209.0455	$C_{10}H_9O_5$	[secoiridoid aglycone core<br>$–CH_3OH–H]^–$	8	**1**, **6**, **14**, **16**, **23**(**10**), **28**, **32**, **33**	
213.0408	213.0405	$C_9H_9O_6$	$[M–C_{14}H_{24}O_{10}–H]^–$	2	**1** and **15** only	
213.0770 (a)	213.0768	$C_{10}H_{13}O_5$	$[M–C_6H_{10}O_5–H]^–$<br>$[M–C_6H_{10}O_5–C_8H_8O_2\text{-}CO_2–H]^–$	2	loganic acid (**8**)<br>**17**	molecules 2016 21
223.0615	223.0612	$C_{11}H_{11}O_5$	[secoiridoid core$–H_2O–H]^–$	18	*absent* from **6**, **14**, **16**, **17**, **21**, **22**, **28**, **32**<br>present in stds **9**, **10**, **11**	5, 7, 17
239.0561	239.0561	$C_{11}H_{11}O_6$	[secoiridoid core+O$–H_2O–H]^–$	2	**17** and **21**	**7**
241.0730	241.0718	$C_{11}H_{13}O_6$	[secoiridoid aglycone core–H]$^–$	3	base peak in **6**, **28**, **33**	7
259.0978	259.0976	$C_{15}H_{15}O_4$	[ligustroside$–C_{10}H_{16}O_8–H]^–$	6	**2**, **18**, **20**, **30**, **31**, **4**	5, 7
265.0726	265.0718	$C_{13}H_{13}O_6$	$[M–C_{10}H_{20}O_{10}–H]^–$<br>$[M–C_{29}H_{40}O_{16}–H]^–$	2	**15**<br>**29**	
275.0924	275.0925	$C_{15}H_{15}O_5$	[oleuropein$–C_{10}H_{16}O_8–H]^–$	2	**21, 23**	5, 7, 17
291.0878	291.0874	$C_{15}H_{15}O_6$	[ligustroside$–C_{10}H_{16}O_6–H]^–$	7	**2**, **18**, **20**, **21**, **30**, **31**, **4**	5, 7
299.1139	299.1136	$C_{14}H_{19}O_7$	[salidroside–H]$^–$	2	**2**, **3**<br>nuzhenide(**9**), GL3(**11**)	5, 17
307.0830	307.0823	$C_{15}H_{15}O_7$	[oleuropein$–C_{10}H_{16}O_6–H]^–$	3	oleuropein(**23**/**10**), **17**	5, 7, 17
361.1309	361.1293	$C_{19}H_{21}O_7$	$[M–2C_6H_{10}O_5–H]^–$<br>$[M–2C_6H_{10}O_5–C_{11}H_{12}O_5–H]^–$	5	**2**, **18**, **20**<br>**4** and **31**	5

421.1510	421.1504	$C_{21}H_{25}O_9$	$[M–C_{10}H_{16}O_8–H]^–$<br>$[M–C_{10}H_{16}O_8–C_{17}H_{22}O_{10}–H]^–$	stds	nuzhenide(**9**)<br>GL3(**11**)	17
453.1403	453.1402	$C_{21}H_{25}O_{11}$	$[M–C_{10}H_{16}O_6–H]^–$<br><br>$[M–C_{10}H_{16}O_8–C_{17}H_{22}O_{10}–H]^–$	4	**2**, **3**, **18**, **20** and nuzhenide(**9**)<br>GL3(**11**)	5, 17
523.1842	523.1821	$C_{25}H_{31}O_{12}$	$[M–C_6H_{10}O_5–H]^–$<br>$[M–C_{17}H_{22}O_{10}–H]^–$<br>$[M–C_6H_{10}O_5–C_{17}H_{22}O_{10}–H]^–$	3	nuzhenide(**9**)<br>**31**<br>GL3(**11**)	5, 17
571.2399	571.2402	$C_{27}H_{39}O_{13}$	$[M–C_{16}H_{20}O_{10}–H]^–$<br>$[M–C_{17}H_{22}O_{10}–H]^–$	2	**32**<br>**33**	

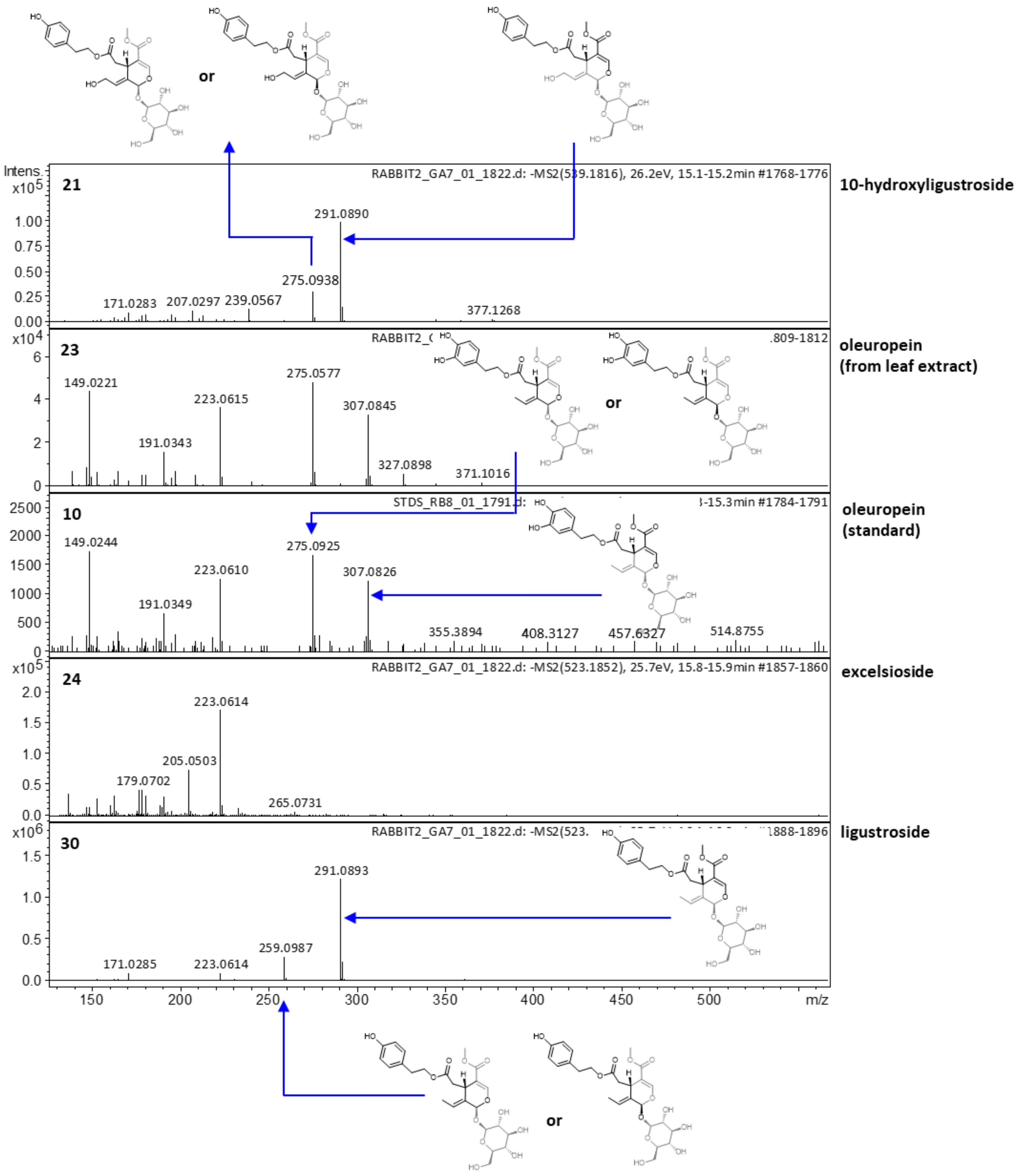

Supplementary Figure S19 – Negative ion mode MS2 spectra of selected secoiridoid glycosides **21**, **23**, **24** and **30** and oleuropein standard **10**. **30** (ligustroside) exhibits the same losses of 232 and 264 as oleuropein, whereas **21** (10-hydroxyligustroside) and **24** (excelsioside) do not.

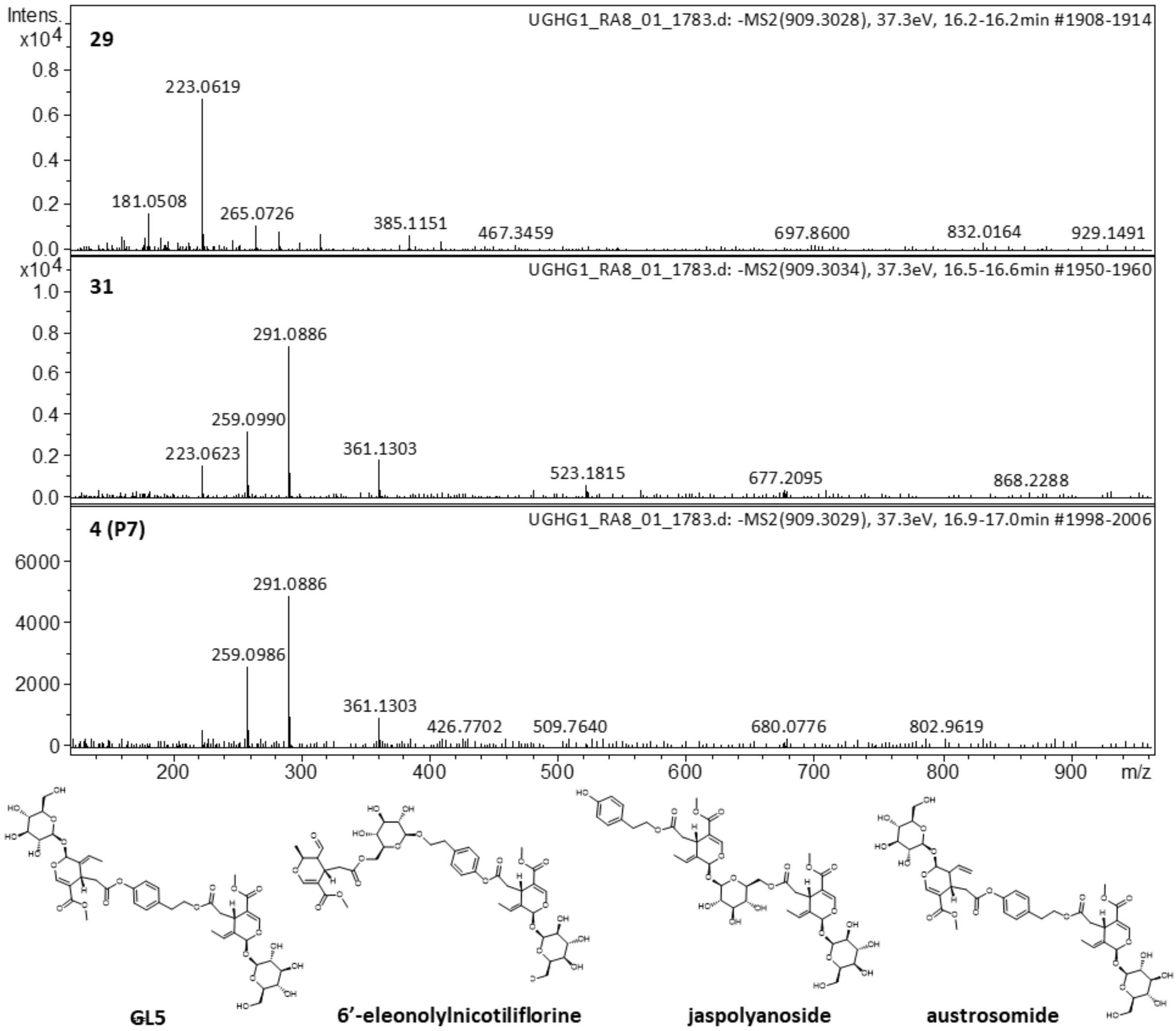

Supplementary Figure S20 – Negative ion mode MS2 spectra of **29**, **31** and **4**(P7). All are isomers of GL5, previously isolated from *F. excelsior*. Structures of previously reported secoiridoid glycosides with formula $C_{42}H_{54}O_{22}$ are also shown.

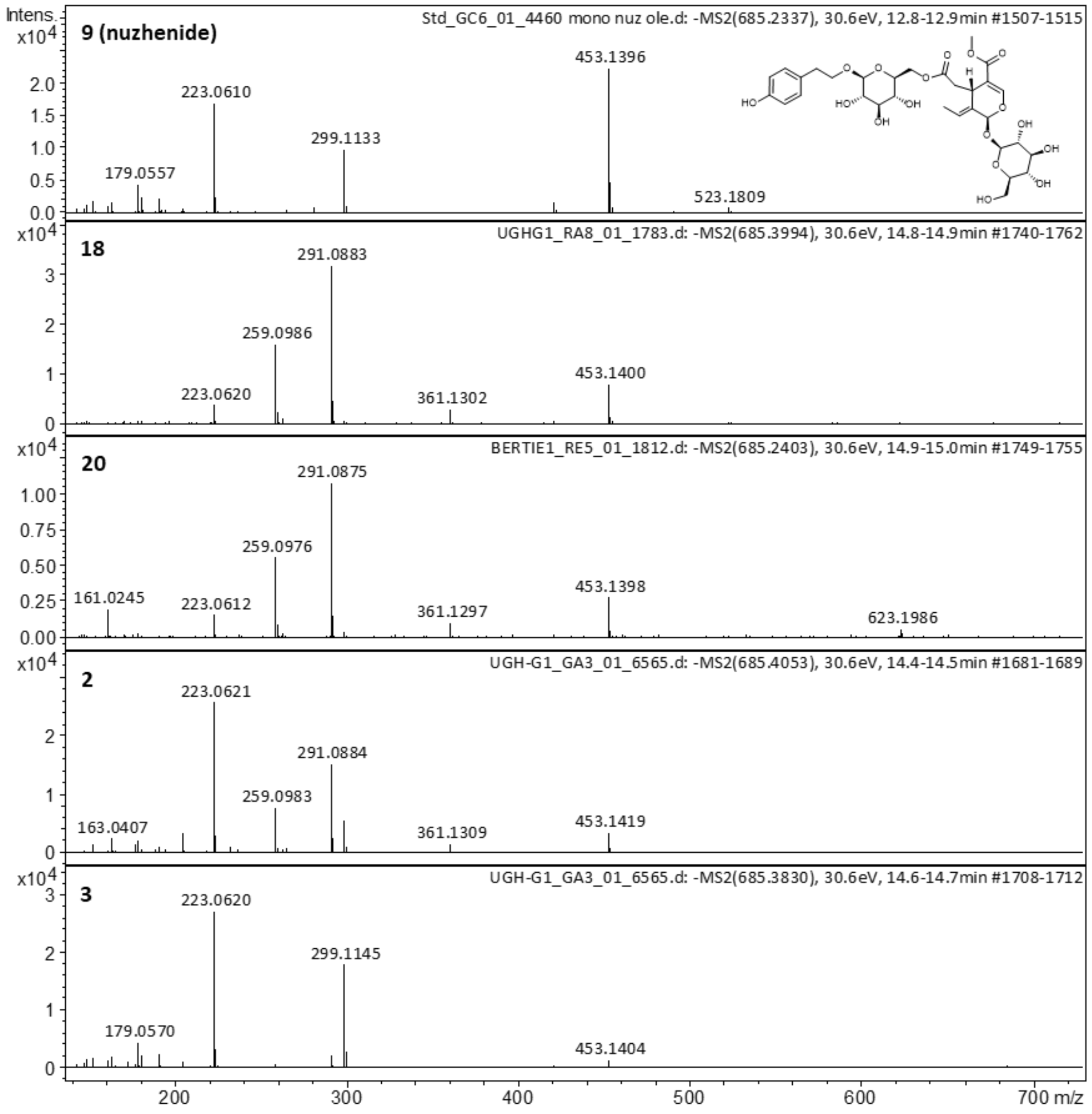

Supplementary Figure S21 – Negative ion mode MS2 spectra of nuzhenide (**9**) and the isomers **2**(P5), **3**(P1/N4), **18** and **20** present in ash leaf extracts analysed in this study.

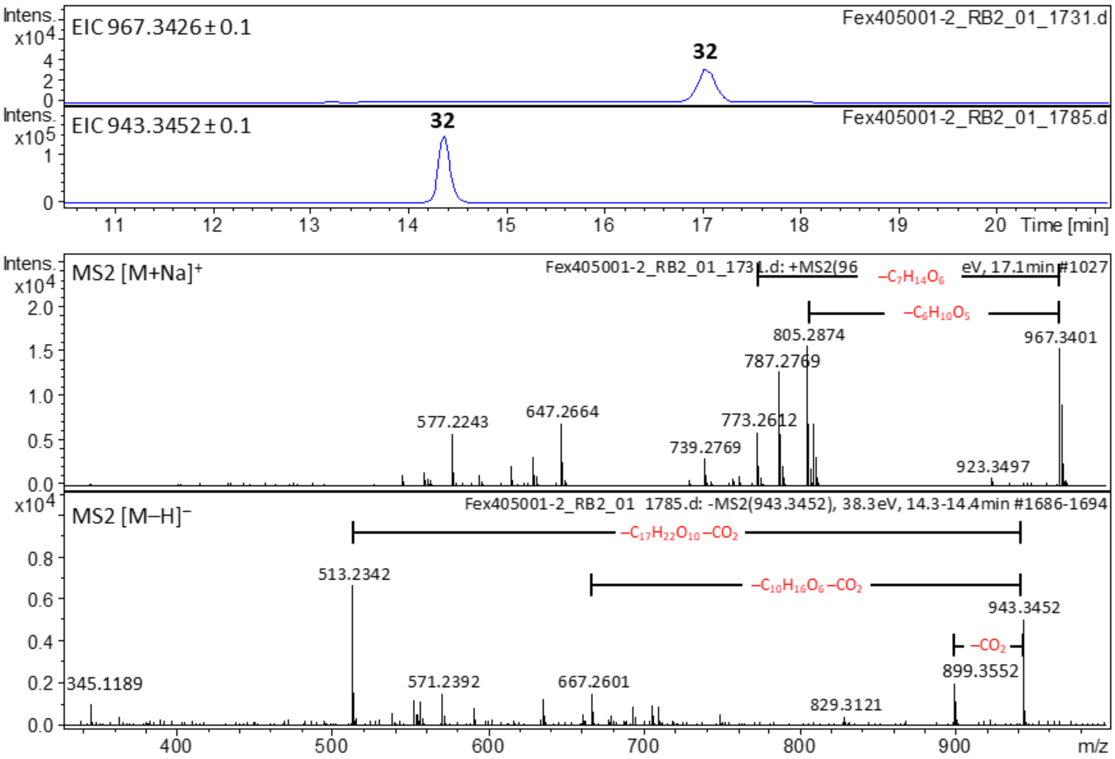

Supplementary Figure S22 – Extracted ion chromatograms of **32** in positive and negative ion modes and MS2 spectra of [M+Na]+ and [M–H]− adducts of **32** demonstrating characteristic losses of 162 ($C_6H_{10}O_5$) and 194 ($C_7H_{14}O_6$) in positive ion mode and losses 276 ($C_{10}H_{16}O_6$+CO_2) and 430 ($C_{17}H_{22}O_{10}$+CO_2) in negative ion mode.

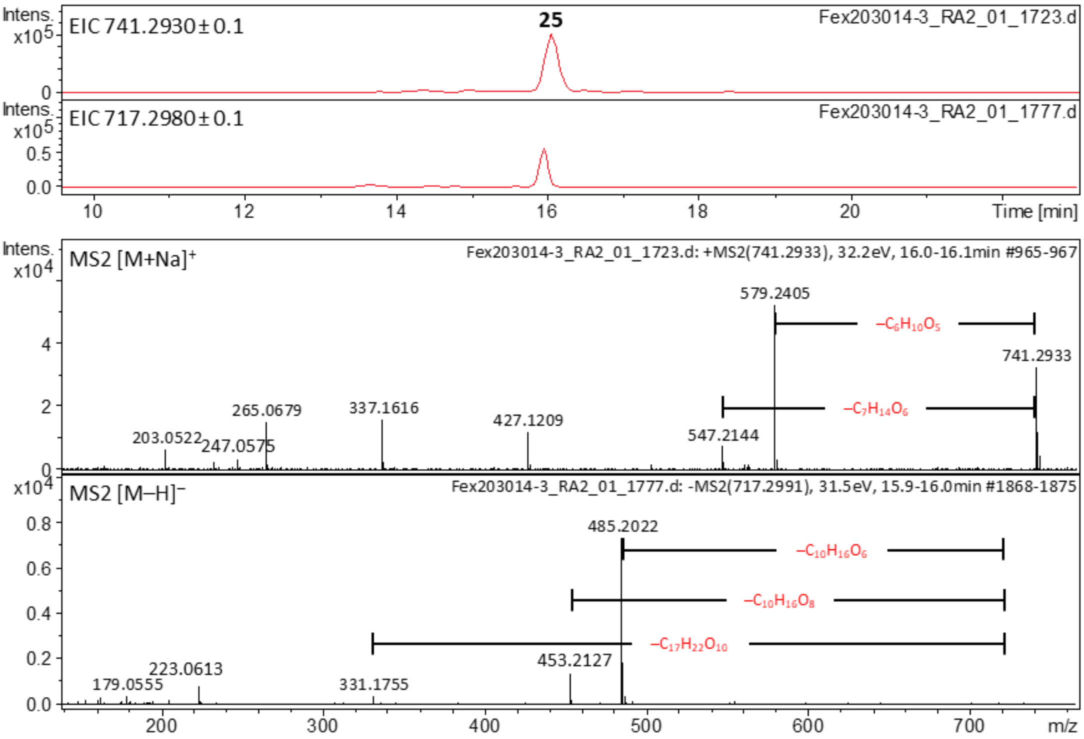

Supplementary Figure S23 – Extracted ion chromatograms of **25** in positive and negative ion modes and MS2 spectra in positive and negative ion modes.

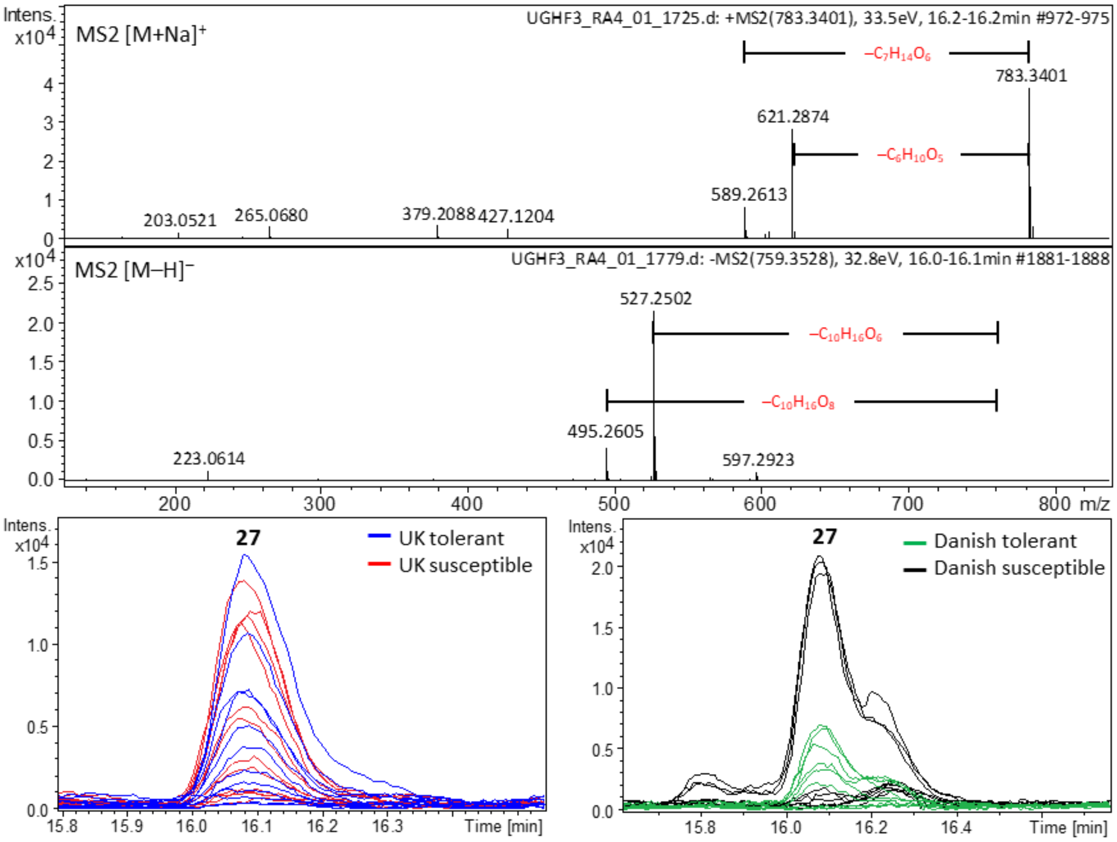

Supplementary Figure S24 – MS2 spectra of [M+Na]$^+$ and [M–H]$^-$ ions of **27** in positive and negative ion modes and extracted ion chromatograms (*m/z* = 783.3405) in positive ion mode showing distribution of 27 in Danish and UK ash.

Supplementary Table S6 **– Statistical analyses (fold change and t-test) of secoiridoids in UK ash leaves associated with tolerance (n = 12) and susceptibility (n = 12) to ash dieback used to generate Figure 4g**

compound	*positive ion mode*					*negative ion mode*				
	m/z *[M+Na]*$^{+}$	*fold change*	*t stat*	*p value*	*chemotype*	m/z *[M–H]*$^{-}$	*fold change*	*t stat*	*p value*	*chemotype*
**25**	741.2935	0.53164	-2.3066	0.030874	SUS>TOL	717.2975	0.45119	-2.4791	0.021316	SUS>TOL
**15**	589.1729	0.5414	-1.8553	0.076998	SUS>TOL	565.1774	0.50538	-1.8693	0.074948	SUS>TOL
**1**	589.1728	0.31526	-1.8511	0.077634	SUS>TOL	565.1774	0.24021	-1.9121	0.068976	SUS>TOL
**28**	757.2878	0.1814	-2.1913	0.03931	SUS>TOL	733.2924	0.17863	-2.485	0.062646	SUS>TOL
**32**	967.3426	1.9193	2.1294	0.044655	TOL > SUS	943.3453	1.9733	2.1392	0.04377	TOL>SUS
**29**	933.2989	2.8487	2.0151	0.056272	TOL > SUS	909.3034	2.4055	1.5918	0.12571	TOL>SUS
**26**	625.2092	1.554	1.8438	0.078731	TOL > SUS	601.2138	1.6353	1.9021	0.070337	TOL>SUS
**24**	547.1782	2.0779	1.7979	0.085925	TOL > SUS	523.1821	3.2095	1.284	0.21249	TOL>SUS
p value > 0.1 in both pos and neg modes										
**22**	1033.316	0.61228	-1.4214	0.16922	SUS>TOL	1009.3194	0.33179	-1.2479	0.22517	SUS>TOL
**31**	933.3004	1.7302	1.1886	0.24729	TOL > SUS	909.3034	2.0437	1.3341	0.19582	TOL>SUS

Supplementary Table S7 **– Statistical analyses (fold change and t-test) of secoiridoids in Danish ash leaves associated with tolerance (n = 9) and susceptibility (n = 9) to ash dieback used to generate Figure 4h**

compound	*positive ion mode*					*negative ion mode*				
	m/z *[M+Na]*$^{+}$	*fold change*	*t stat*	*p value*	*chemotype*	m/z *[M–H]*$^{-}$	*fold change*	*t stat*	*p value*	*chemotype*
**2**	709.2311	0.24229	-5.6757	3.44E-05	SUS>TOL	685.2349	0.27567	-4.3773	0.000469	SUS>TOL
**18**	709.2311	0.2707	-4.8469	0.000178	SUS>TOL	685.2349	0.28342	-5.7941	2.74E-05	SUS>TOL
**26**	625.2092	0.55386	-4.2462	0.000616	SUS>TOL	601.2138	0.46972	-4.2474	0.000615	SUS>TOL
**3**	709.2308	0.036123	-4.1359	0.000776	SUS>TOL	685.2349	0.049919	-3.7096	0.001903	SUS>TOL
**1**	589.1728	0.064265	-3.7532	0.001736	SUS>TOL	565.1774	0.12052	-4.5712	0.000314	SUS>TOL
**13**	427.1213	0.57045	-2.9455	0.009499	SUS>TOL	403.1246	0.53313	-1.5404	0.14301	SUS>TOL
**27**	783.3401	0.50909	-2.4835	0.024472	SUS>TOL	759.3445	0.48561	-1.0978	0.28853	SUS>TOL
**29**	933.2989	0.30221	-2.1532	0.046904	SUS>TOL	909.3034	0.18341	-2.592	0.019658	SUS>TOL
**28**	757.2878	0.069395	-1.7874	0.092824	SUS>TOL	733.2924	0.18088	-2.4669	0.094782	SUS>TOL
**14**	not obs.	n/a	n/a	n/a	n/a	525.1606	0.63451	-1.8387	0.08459	SUS>TOL
p value > 0.1 in both pos and neg modes										
**12**	443.1157	1.6922	1.6808	0.11222	TOL>SUS	not obs.	n/a	n/a	n/a	n/a
**31**	933.3004	0.37913	-1.6543	0.11755	SUS>TOL	909.3034	0.52529	-1.6042	0.12822	SUS>TOL
**4**	933.2987	0.30496	-1.501	0.15283	SUS>TOL	909.3034	0.35263	-1.2866	0.21655	SUS>TOL

Supplementary Table S8 **– Fold change (threshold 1.5) and t test to demonstrate abundance of compounds** 1-34 **between Danish (n = 18) and UK (n = 24) ash leaf extracts used to generate Figure 5a**

compound	*positive ion mode*				*negative ion mode*			
	fold change	*t.stat*	*p.value*	*chemotype*	*fold change*	*t.stat*	*p.value*	*chemotype*
**12**	5.6093	5.7951	9.18E-07	Dan>UK	not obs.	n/a	n/a	n/a
**14**	not obs.	n/a	n/a	n/a	0.4523	-2.1155	0.040663	UK>Dan
**15**	0.18606	-4.1345	0.000177	UK>Dan	0.37014	-2.8982	0.006064	UK>Dan
**16**	not obs.	n/a	n/a	n/a	0.31963	-3.2089	0.002626	UK>Dan
**18***	98.638	6.3568	1.49E-07	Dan>UK	32.92	6.8248	3.29E-08	Dan>UK
**19**	0.058929	-2.6046	0.012851	UK>Dan	0.065155	-3.6082	0.000847	UK>Dan
**20***	0.0021089	-5.5835	1.82E-06	UK>Dan	0.023637	-4.3016	0.000106	UK>Dan
**2***	582.64	6.3961	1.31E-07	Dan>UK	41.245	6.065	3.83E-07	Dan>UK
**3**	2.3421	1.7413	0.089315	Dan>UK	3.3073	2.4114	0.020573	Dan>UK
**22**	0.62071	-1.7867	0.08156	UK>Dan	0.49157	-1.0643	0.29356	
**23**	0.32905	-8.5703	1.36E-10	UK>Dan	0.26276	-6.2796	1.91E-07	UK>Dan
**24**	0.032872	-4.089	0.000203	UK>Dan	0.01223	-2.0573	0.046217	UK>Dan
**25**	0.50345	-2.7242	0.00951	UK>Dan	0.48244	-2.4823	0.01735	UK>Dan
**26**	1.8981	4.2323	0.000131	Dan>UK	2.3991	4.7748	2.42E-05	Dan>UK
**28**	0.34001	-1.5585	0.127		0.15725	-2.6689	0.062584	UK>Dan
**29**	2.2624	2.0682	0.045132	Dan>UK	0.79558	-0.54464	0.58902	
**30**	0.15803	-15.345	2.34E-18	UK>Dan	0.091564	-12.604	1.64E-15	UK>Dan
**31**	0.093991	-3.4318	0.001406	UK>Dan	0.068774	-3.0736	0.003799	UK>Dan
**33**	0.35489	-3.1382	0.003188	UK>Dan	0.28286	-3.9481	0.000311	UK>Dan
**34**	0.27262	-3.3561	0.001742	UK>Dan	0.41589	-3.1883	0.002779	UK>Dan

***18** was observed in 18/18 Danish samples and only 5 UK samples; **20** was observed in 24/24 UK samples and only 7 Danish samples; **2** was observed in 18/18 Danish samples and only 5 UK samples.

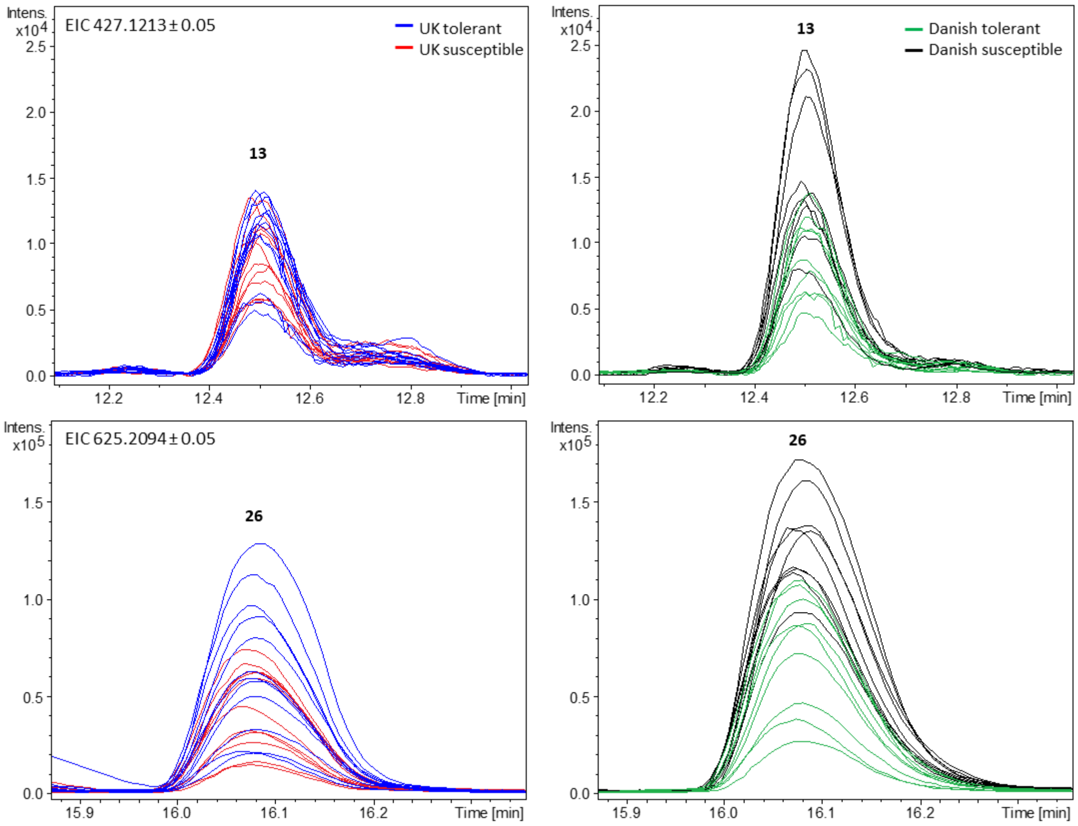

Supplementary Figure S25 – Extracted ion chromatograms of compounds **13** and **26** in positive ion mode showing their distributions in UK and Danish leaf extracts.

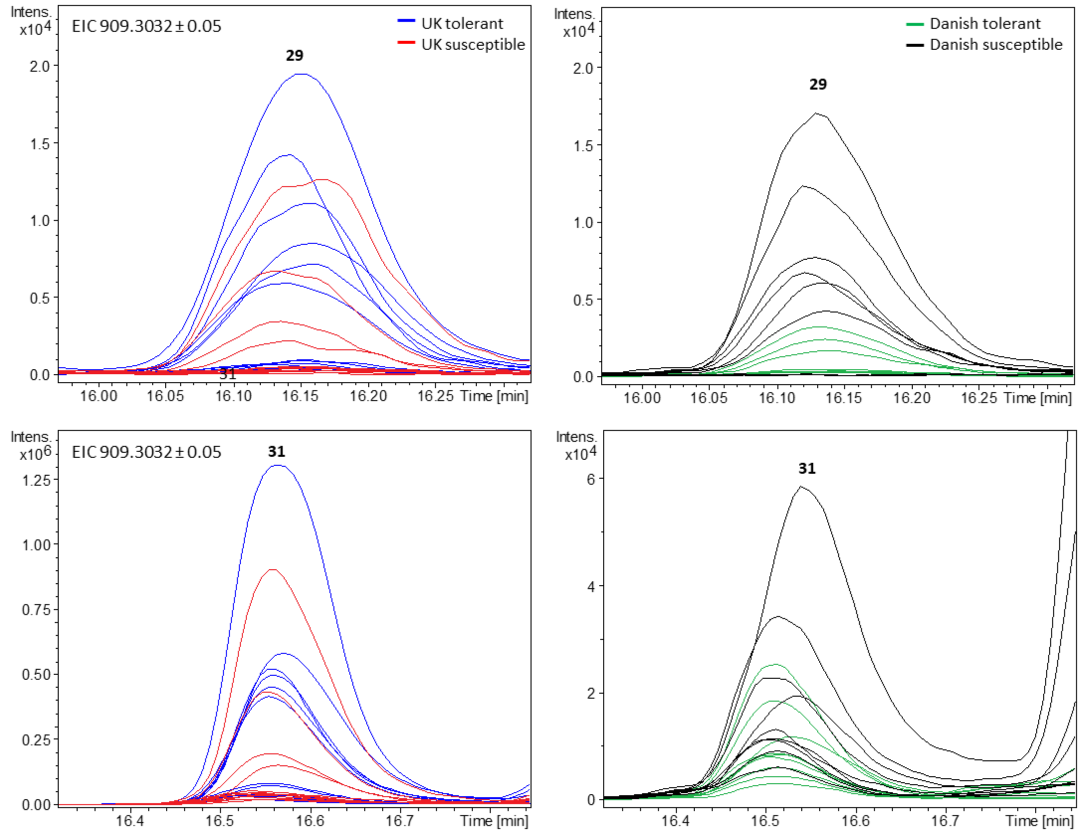

Supplementary Figure S26 – Extracted ion chromatograms of **29** and **31** in positive ion mode showing their distributions in UK and Danish leaf extracts.

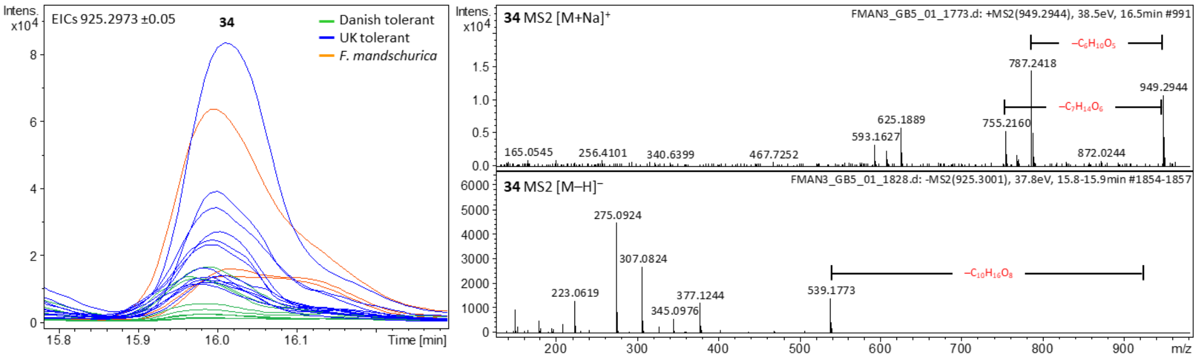

Supplementary Figure S27 – Extracted ion chromatograms (negative ion mode) and MS2 spectra of $[M+Na]^+$ and $[M–H]^-$ ions of **34** in positive and negative ion modes.

References

1. Sollars, E. S. A. *et al.* Genome sequence and genetic diversity of European ash trees. *Nature* **541**, 212–216 (2017).

2. Shen, Y., Lin, C., Chen, C. & Index--jasminum, K. W. Secoiridoid glycosides from Jasminum multiflorum. *Phytochemistry* **29**, 2905–2912 (1990).

3. Damtoft, S., Franzyk, H. & Jensen, S. R. Excelsioside, a secoiridoid glucoside from Fraxinus excelsior. *Phytochemistry* **31**, 4197–4201 (1992).

4. Kiss, A. K., Mańk, M. & Melzig, M. F. Dual inhibition of metallopeptidases ACE and NEP by extracts, and iridoids from Ligustrum vulgare L. *J. Ethnopharmacol.* **120**, 220–225 (2008).

5. Kiss, A. K., Michalak, B., Patyra, A. & Majdan, M. UHPLC-DAD-ESI-MS/MS and HPTLC profiling of ash leaf samples from different commercial and natural sources and their in vitro effects on mediators of inflammation. *Phytochem. Anal.* **31**, 57–67 (2020).

6. Cleary, M. R. *et al.* Genotypes of Fraxinus excelsior with different susceptibility to the ash dieback pathogen Hymenoscyphus pseudoalbidus and their response to the phytotoxin viridiol - A metabolomic and microscopic study. *Phytochemistry* **102**, 115–125 (2014).

7. Song, J., Zhao, L., Rui, W., Guo, J. & Feng, Y. Identification and fragmentation pattern analysis of iridoid glycosides from Fructus ligustri lucidi by UPLC/ESI-QTOF-MS. *J. Liq. Chromatogr. Relat. Technol.* **37**, 1763–1770 (2014).

8. Takenaka, Y., Tanahashi, T., Taguchi, H., Nagakura, N. & Nishi, T. Nine new secoiridoid glucosides from Jasminum nudiflorum. *Chem. Pharm. Bull.* **50**, 384–389 (2002).

9. Tanahashi, T. *et al.* Six secoirioid glucosides from Jasminum polyanthum. *Chem. Pharm. Bull.* **45**, 367–372 (1997).

10. Shen, Y. C., Lin, S. L. & Chein, C. C. Jaspolyside, a secoiridoid glycoside from Jasminum polyanthum. *Phytochemistry* **42**, 1629–1631 (1996).

11. Tanahashi, T. *et al*. Structure Elucidation of Six Acylated Iridoid Glucosides from Jasminum hemsleyi. *Chem. Pharm. Bull.* **43**, 729–733 (1995).

12. Takenaka, Y. *et al.* Secoiridoid glucosides from Fraxinus americana. *Phytochemistry* **55**, 275–284 (2000).

13. Liu, Y. F. *et al.* Hepatoprotective iridoid glycosides from the roots of Rehmannia glutinosa. *J. Nat. Prod.* **75**, 1625–1631 (2012).

14. Egan, P. *et al.* GI 5, a dimer of oleoside, from Fraxinus excelsior (Oleaceae). *Biochem. Syst. Ecol.* **32**, 1069–1071 (2004).

15. Kitagawa, I. *et al*. Pulosarioside, a new bitter trimeric-iridoid diglucoside, from an Indonesian jamu, the bark of Alyxia reinwardtii bl. (Apocynaceae). *Chem. Pharm. Bull.* **36**, 4232–4235 (1988).

16. Takenaka, Y., Tanahashi, T., Nagakura, N. Eight Minor Secoiridoid Glucosides with a Linear Monoterpene Unit from Jasminum polyanthum. *Chem. Pharm. Bull.* **46**, 1776–1780 (1998).

17. Zhong, X. *et al.* Metabolomics approach based on ultra-high-performance liquid chromatography coupled with quadrupole-time-of-flight mass spectrometry to identify the chemical constituents of the Traditional Chinese Er-Zhi-Pill. *J. Sep. Sci.* **40**, 2713–2721 (2017).

18. Kucharska, A. Z. & Fecka, I. Identification of iridoids in edible honeysuckle berries (lonicera caerulea L. var. kamtschatica Sevast.) by UPLC-ESI-qTOF-MS/MS. *Molecules* **21**, 1157 (2016).
